# Optimizing progeny allocation strategies in breeding schemes while updating genomic prediction models

**DOI:** 10.1270/jsbbs.25056

**Published:** 2026-06-25

**Authors:** Kosuke Hamazaki, Koji Tsuda, Hiroyoshi Iwata

**Affiliations:** 1 RIKEN Center for Advanced Intelligence Project (AIP), RIKEN, 178-4-4 Wakashiba, Kashiwa, Chiba 277-0871, Japan; 3 Graduate School of Frontier Sciences, The University of Tokyo, 5-1-5 Kashiwa-no-ha, Kashiwa, Chiba 277-8561, Japan; 4 Graduate School of Agricultural and Life Sciences, The University of Tokyo, 1-1-1 Yayoi, Bunkyo, Tokyo 113-8657, Japan

**Keywords:** genomic selection, breeding schemes, optimization, progeny allocation, estimated marker effects, model updates

## Abstract

Genomic selection has revolutionized breeding by enabling early identification of superior individuals using genome-wide markers. Over the past decade, new selection and mating strategies leveraging optimization methods have been introduced to improve decision-making in breeding programs. However, optimizing breeding remains challenging when the positions and effects of quantitative trait loci are unknown. We developed a framework that optimizes breeding strategies while updating genomic prediction models during recurrent selection programs. In these programs, selection, crossing, and progeny allocation are repeated across generations under limited population sizes. By updating prediction models at intermediate generations, we re-optimized how progeny are allocated among crosses, i.e., the number of progeny assigned to each cross under a fixed total population size, based on newly accumulated data. Our simulations compared this approach with equal allocation and optimal cross selection methods across various conditions. The optimized allocation strategy significantly outperformed other approaches under moderate to low selection intensities, particularly when combined with model updates. While genetic gains plateaued without updates, our approach with updates enabled continuous improvement through the fourth generation of recurrent selection. The framework showed robustness across different conditions and maintained genetic diversity, confirming its effectiveness under estimated marker effects intended for real-world breeding programs.

## Introduction

As the global population grows, the demand for stable food supply increases, while climate change complicates food production (Godfray *et al.* 2010, Lobell *et al.* 2011, Wheeler and von Braun 2013). This creates an urgent need to efficiently develop new crop cultivars with desired agricultural features, including environmental adaptability, high yield, and nutritional value (Ray *et al.* 2013, Tilman *et al.* 2011). Conventional breeding methods cannot address these challenges quickly enough, necessitating technological innovations (Bailey-Serres *et al.* 2019, Tester and Langridge 2010, Varshney *et al.* 2021). In recent years, genomic selection (GS) has emerged as a breakthrough method that significantly enhances breeding efficiency (Meuwissen *et al.* 2001). GS utilizes whole-genome DNA marker information and previously trained genomic prediction (GP) models to predict genotypic values before phenotypic evaluation, enabling early selection of superior individuals (Crossa *et al.* 2017, Jannink *et al.* 2010, Meuwissen *et al.* 2001). This approach shortens breeding cycles, allowing for significant genetic gains in a short period (Heffner *et al.* 2009). GS has proven particularly valuable for improving complex quantitative traits such as environmental stress tolerance, yield, and nutritional value across numerous crop species (Bernardo and Yu 2007, Duhnen *et al.* 2017, Heffner *et al.* 2011a, Minamikawa *et al.* 2017, Onogi *et al.* 2015, Yabe *et al.* 2018).

Since GS was introduced to crop breeding, numerous studies have aimed to improve selection efficiency through the enhancement of GP models (de los Campos *et al.* 2009, Gianola and van Kaam 2008, Gianola *et al.* 2011, Habier *et al.* 2011, Heffner *et al.* 2011b, Jannink *et al.* 2010, Meuwissen *et al.* 2001). However, effectively implementing GS in breeding schemes spanning multiple generations requires not only better GP models but also well-designed selection and mating strategies based on GP results (Wang *et al.* 2018). For selection strategies, instead of simply using predicted breeding values by GP (GEBV) as in Meuwissen *et al.* (2001), methods such as weighted breeding value (WBV) (Goddard 2009, Jannink 2010), optimal haploid value (OHV) (Daetwyler *et al.* 2015), optimal population value (OPV) (Goiffon *et al.* 2017), predicted cross value (PCV) (Han *et al.* 2017), and expected haploid maximum breeding value (EMBV) (Müller *et al.* 2018) have been devised that consider allele frequency and recombination to provide mid- to long-term advantages. Additionally, for mating strategies, several approaches have been proposed that directly identify superior crossing pairs by evaluating potential genetic variance in the progeny of crossed pairs through simulations (Lian *et al.* 2015, Mohammadi *et al.* 2015, Yao *et al.* 2018) or theoretical formulas (Allier *et al.* 2019b, Lehermeier *et al.* 2017, Zhong and Jannink 2007).

In recent years, optimization methods for various decisions in breeding schemes have gained significant attention (Diot and Iwata 2022, Hamazaki and Iwata 2022, Hassanpour *et al.* 2025, Jannink *et al.* 2023, Moeinizade *et al.* 2022). For selection and mating strategies, optimal contribution selection, an early approach, optimizes the contribution of each genotype to the next generation by balancing genotypic gains with genetic diversity based on the pedigree information (Meuwissen 1997). This method evolved to incorporate genomic information (Kemper *et al.* 2012) and developed into optimal cross selection (OCS), which selects ideal crossing pairs while balancing genotypic gains with genetic diversity (Kinghorn 2011), with practical applications of OCS also being proposed (Allier *et al.* 2019b, 2020, Gorjanc *et al.* 2018). While research on mating design problem has a long history (Jansen and Wilton 1985), the past decade has seen numerous new methods emerge alongside OCS, mainly driven by the utilization of genomic information and advancements in simulation technologies. Look-ahead selection predicts the genetic gains of new varieties produced several generations later under simplified assumptions to select optimal mating pairs (Moeinizade *et al.* 2019, Zhang and Wang 2022). Cross potential selection combines estimated distribution of genotypic values for inbred progeny with integer programming to select multiple crossing pairs simultaneously under some constraints (Sakurai *et al.* 2024). Other approaches determine the optimal progeny allocations across mating pairs to achieve a Pareto front of expected genetic gain and variance in the next generation (Hunter and McClosky 2016). Notably, Hamazaki and Iwata successfully integrated breeding simulations with black-box optimization to maximize the genetic gains of new varieties over multiple generations by optimally allocating progeny for each mating pair at each generation (Hamazaki and Iwata 2024), and subsequently enhanced this approach by incorporating automatic differentiation, a state-of-the-art computational technology (Hamazaki *et al.* 2025).

However, many studies on decision-making in breeding programs often operate under the ideal assumption that true marker effects are known (Amini *et al.* 2021, Daetwyler *et al.* 2015, Goiffon *et al.* 2017, Hamazaki and Iwata 2024, Hamazaki *et al.* 2025, Kemper *et al.* 2012, Moeinizade *et al.* 2019, 2022, Wang *et al.* 2018, Zhang and Wang 2022). In other words, these studies propose breeding strategies assuming that the positions and effects of genes controlling traits can be completely understood. In reality, it is practically impossible to fully grasp the exact positions and effects of quantitative trait loci (QTLs) in advance, creating a significant disconnect between the above assumptions and the reality of actual breeding programs. Although fully identifying loci and effects for quantitative traits remains challenging even in major crops such as rice and maize, QTLs and genes have been identified for some traits and used in breeding through QTL pyramiding and marker-assisted selection (Collard and Mackill 2008). For less-studied crops, such as neglected and underutilized species (NUS) (Padulosi *et al.* 2013), however, such discoveries are rare even for traits governed by just a few major genes, thereby broadening the range of traits where GP is particularly valuable. Therefore, evaluating breeding strategies based on marker effects estimated through GP has significant value, particularly for improving breeding efficiency in less-studied crops.

GP accuracy directly impacts selection efficiency, making it crucial when advancing breeding schemes that use marker effects estimated by GP. Generally, as breeding schemes progress and selection continues, alleles become fixed, and the genetic variance of the population decreases significantly due to the Bulmer effect (Bulmer 1971, Van Grevenhof *et al.* 2012). This causes later generations to differ substantially from the initial population. When using a GP model trained only on the initial population to predict genotypic values of these later generations, prediction accuracy gradually declines with each generation. To maintain GP accuracy, it is necessary to update the GP model regularly throughout the breeding scheme. In fact, previous studies have demonstrated that these periodic model updates enable continuous genetic improvement, even in mid-term or long-term breeding schemes (Gorjanc *et al.* 2018, Jannink 2010).

Based on this background, our study proposes a framework for optimizing breeding strategies while updating the GP model during the breeding scheme, assuming realistic situations where the QTL positions and effects remain unknown. Specifically, we focus on the approach developed by Hamazaki and Iwata (2024) for optimizing progeny allocation across mating pairs, and verify its effectiveness by re-optimizing the progeny allocation strategy in conjunction with model updates.

## Materials and Methods

Throughout the study, R version 4.4.1 (R Core Team 2024) was used to simulate the marker genotype of the initial breeding population, implement breeding optimization, and conduct breeding simulations. We have also provided a glossary table of the variables that appear in this section to help readers understand the framework proposed in this study (Supplemental Table 1).

### System design of the study

As outlined by Hamazaki and Iwata (2024), breeders follow a breeding scheme based on the progeny allocation strategy proposed by an “AI breeder” (Supplemental Fig. 1). The AI breeder receives current breeding population data—including marker genotype data, phenotypic data of target traits, and recombination rates between genetic markers—from actual breeders and outputs a set of parameters representing the optimal allocation strategy. Below, we describe how the AI breeder optimizes the resource allocation of progeny.

After receiving the current population information at generation t, the AI breeder explores the optimal allocation strategy through repeated breeding simulations based on different allocation parameters, ${\left\{{𝛉}^{\left(\tau \right)}\right\}}_{\tau =\tau_{0},\ldots ,T_{s}-1}$ (Supplemental Fig. 1). The parameter vector ${𝛉}^{\left(\tau \right)}\in {\mathbb{R}}^{d}$ serves as the AI breeder’s decision-making framework. In these simulations, $\tau \in \left\{\tau_{0},\ldots ,T_{s}-1\right\}$ denotes the current generation number, where $\tau_{0}\in \mathbb{N}$ and $T_{s}\in \mathbb{N}$ are the initial and final generation numbers. On the other hand, $t\in \left\{0,\ldots ,T_{r}-1\right\}$ denotes the current generation number in breeding schemes, which are conducted by actual breeders, where $T_{r}\in \mathbb{N}$ is the final generation in actual breeding schemes.

In each breeding simulation, we assess the performance of allocation strategies by computing the genetic gain in the final generation $T_{s}$ after multiple cycles of selection, mating, and allocation. The genetic gain, $g^{\left(T_{s}\right)}\in \mathbb{R}$, quantifies the improvement in genotypic values of the top 5% of individuals in the final population compared to the initial population. Thus, each breeding simulation can be represented as a function with input ${\left\{{𝛉}^{\left(\tau \right)}\right\}}_{\tau =\tau_{0},\ldots ,T_{s}-1}$ and output $g^{\left(T_{s}\right)}$, as in Equation 1.


$$g^{\left(T_{s}\right)}=F\left(\left\{{𝛉}^{\left(\tau \right)}\right\},\left\{{𝛈}^{\left(\tau \right)}\right\}\right),$$ (1)

where F is a function representing each breeding simulation. Because breeding simulations incorporate random factors related to recombination events when generating new individuals from mating pairs in each generation τ, represented by random variables ${𝛈}^{\left(\tau \right)}\in {\mathbb{R}}^{M}$ in this study. To evaluate the performance of $\left\{{𝛉}^{\left(\tau \right)}\right\}$, we must calculate the expectation with respect to $\left\{{𝛈}^{\left(\tau \right)}\right\}$ through repeated breeding simulations. Thus, the objective of the AI breeder is to optimize $\left\{{𝛉}^{\left(\tau \right)}\right\}$ so that $F\left(\left\{{𝛉}^{\left(\tau \right)}\right\}\right)$ in the following Equation 2 is maximized.


$$F\left(\left\{{𝛉}^{\left(\tau \right)}\right\}\right)={𝔼}_{𝛈}\left[g^{\left(T_{s}\right)}\right]={𝔼}_{𝛈}\left[F\left(\left\{{𝛉}^{\left(\tau \right)}\right\},\left\{{𝛈}^{\left(\tau \right)}\right\}\right)\right].$$ (2)

As noted by Hamazaki and Iwata (2024), this problem can be viewed as a black-box optimization problem where only the inputs and outputs of function F are observable. Therefore, we optimize the allocation strategy by combining repeated breeding simulations with StoSOO (Valko *et al.* 2013), a black-box optimization technique.

Here, since the GP model used in breeding simulations conducted by the AI breeder becomes less accurate over generations, it requires updates during the breeding scheme. Thus, in this study, we update the GP model at generation $t=t_{u}$ ($0<t_{u}<T_{r}$), at which point the AI breeder re-optimizes the allocation strategy (Fig. 1A). Specifically, the construction of the GP models and the optimization by the AI breeder occur twice—at generation t=0 and $t=t_{u}$. Throughout the study, we set $T_{r}=4$ and $t_{u}=2$. This approach allows us to examine how model updates affect the allocation strategy optimization by comparing results with and without model updates (Fig. 1A, 1B). We compared the optimized allocation strategy proposed by Hamazaki and Iwata (2024) with equal allocation and OCS under the breeding schemes with or without model updates.

### Simulation of the initial breeding population and genome structure

The marker genotype and QTLs of the initial breeding population were simulated following Hamazaki and Iwata (2024). Using the coalescent simulator GENOME (Liang *et al.* 2007), we first generated candidate loci for genome-wide markers and QTLs of founder haplotypes. We assumed a virtual diploid crop with ten chromosomes (2n=20), with 500 loci (including both markers and QTLs) on each chromosome. First, we independently generated 4,000 founder haplotypes per chromosome using GENOME with these parameters: “-pop 1 4000 -N 4000 -c 1 -pieces 100000 -rec 0.0001 -s 4000 -maf 0.01 -tree 0 -mut 0.00000001”. From these 4,000 loci, we randomly selected 500 loci with a minor allele frequency (MAF) ≥0.01. We then created a historical population of 2,000 founder genotypes by sampling two haplotypes with replacement from all founder haplotypes, as implemented in the R package “BreedingSchemeLanguage” by Yabe *et al.* (2017). This historical population then underwent random mating for 300 generations, followed by selecting 200 genotypes that were randomly mated for 15 generations under the assumption of a population bottleneck to create extensive linkage disequilibrium. Finally, we expanded the population to 2,000 genotypes and conducted random mating for three generations to produce 2,000 pre-breeding materials, following the methodology of Müller *et al.* (Müller *et al.* 2017, 2018).

Following the creation of pre-breeding materials, we selected N=250 representative genotypes for the initial breeding population to capture the full genetic diversity of the pre-breeding materials, as described in Hamazaki and Iwata (2024). To ensure comprehensive representation, we employed the k-medoids clustering method using the “pam” function of the R package “cluster” version 2.1.2 (Maechler *et al.* 2021). This approach divided the pre-breeding materials into 250 distinct groups based on their genotype scores, from which we selected the medoid of each group as a representative genotype. Throughout this study, genotype scores were coded as: 0 for homozygous reference alleles, 1 for heterozygous alleles, and 2 for homozygous alternative alleles. The resulting initial population had a relatively small number of genotypes, which is typical of small-scale breeding schemes where strategy optimization has a significant impact. This sample size of 250 was determined based on several previous studies analyzing genomic breeding in NUS (Akohoue *et al.* 2020, Jones *et al.* 2025).

### Simulation of QTLs and phenotypic values

In simulating QTLs and phenotypes, we assumed one quantitative trait with a simple genetic architecture as a target trait. First, from the 500 loci simulated in the previous subsection, we randomly selected q loci per chromosome as QTLs. Here, we prepared two scenarios with different numbers of QTLs (i.e., q=3 in Scenario 1 and q=60 in Scenario 2). For simplicity, all QTL effects were additive and independently sampled from the same normal distribution. Then, we calculated true additive genotypic values (breeding values) for the initial population by multiplying QTL genotype scores by their effects and summing the products (Meuwissen *et al.* 2001). We then generated phenotypic values by adding random errors to these genotypic values. These errors were independently sampled from the identical normal distribution, whose variance was determined assuming a heritability of 0.7 throughout the study. While a heritability of 0.7 may appear somewhat high, changes in heritability primarily affect the accuracy of the GP model, and it has little significant impact on the relative ranking of allocation strategies (Jannink 2010, Sakurai *et al.* 2024). We therefore set a slightly higher heritability within a realistic range to make the differences among strategies clearly visible.

### Genomic prediction models and selection criteria

In this study, the AI breeder could not directly access QTL information or true genotypic values. Instead, it constructed a GP model using genome-wide markers and phenotypic data to estimate marker effects. Thus, we randomly selected 400 non-QTL loci per chromosome as markers (totaling M=4,000 markers) to build this GP model. In this study, a Bayesian linear regression model in Equation 3 was used as the GP model (Meuwissen *et al.* 2001).


$${𝐲}^{\left(t\right)}=𝟏\mu +{𝐖}^{\left(t\right)}𝛂+{𝐞}^{\left(t\right)},$$ (3)

where ${𝐲}^{\left(t\right)}\in {\mathbb{R}}^{N}$ is a vector of phenotypic values at generation t, $𝟏\in {\mathbb{R}}^{N}$ is a vector that vertically aligns 1, μ∈ℝ is an intercept, ${𝐖}^{\left(t\right)}\in {\left\{0,1,2\right\}}^{N\times M}$ is a matrix of marker genotype scores at generation t, $𝛂\in {\mathbb{R}}^{M}$ is a vector of marker effects, and ${𝐞}^{\left(t\right)}\in {\mathbb{R}}^{N}$ is an error term. Here, by appropriately designing the prior distribution of marker effects, various Bayesian linear regression models can be implemented. For this study, we used the BayesB model (Heffner *et al.* 2011b, Meuwissen *et al.* 2001) for Scenario 1 with a small number of QTLs and the Bayesian Ridge Regression model (Meuwissen *et al.* 2001) for Scenario 2 with a large number of QTLs. These models were implemented using the R package “BGLR” version 1.0.8 (Pérez and de los Campos 2014).

### Breeding schemes with and without model updates

The breeding scheme assumed in this study closely followed the one described by Hamazaki and Iwata (2024), except for the updates to GP models and allocation strategies conducted during the breeding process. For simplicity and due to computational limitations, we consistently employed a small-scale breeding scheme with simple recurrent genomic selection targeting minor crops such as NUS. Note that in this study, we assume cross-pollinated and vegetatively propagated crops, and do not perform selfing aimed at genotype fixation toward the final generation. The key difference from previous research was that this study contained several discrepancies between the breeding schemes intended for practical breeding (Fig. 2) and those simulated by the AI breeder for the optimization of progeny allocation (Supplemental Fig. 2).

#### Set initial and final generation numbers

To start a breeding scheme, we set the current and final generation numbers as t=0 and $T_{r}=4$ for the scheme intended for practical breeding (Fig. 2).

#### Construct GP models and optimize progeny allocation strategies

At generation t=0 in the scheme implemented by actual breeders, marker effects were estimated based on the marker genotype data and phenotypic data of the initial population, as described in the subsection *Genomic prediction models and selection criteria*. The progeny allocation strategies for subsequent generations were also optimized based on this initial population information, as outlined in the subsection *Optimization of progeny allocation strategies*. For breeding schemes with model updates, both GP model construction and optimization of allocation strategies were conducted again at generation $t=t_{u}$, where $t_{u}=2$ throughout the study (Figs. 1A, 2). In contrast, for the comparative breeding scheme without model updates, the AI breeder optimized progeny allocation starategy only once, at the initial generation (t=0) (Fig. 1B).

#### Select parent candidates

From the current population at generation t, we selected n genotypes as parent candidates for mating based on the increasing order of weighted breeding values (WBVs), ${𝐮}^{\left(\text{WBV}\right)\left(t\right)}$, as given in Equation 4. WBVs are variants of breeding values designed to achieve better gains in long-term programs by emphasizing rare alleles and thus maintaining genetic diversity (Jannink 2010).


$${𝐮}^{\left(\text{WBV}\right)\left(t\right)}=\sum_{m=1}^{M}{𝐰}_{m}^{\left(t\right)}{\hat{\alpha }}_{m}{p_{m}^{\left(t\right)}}^{-\frac{1}{2}},$$ (4)

where ${𝐰}_{m}^{\left(t\right)}\in {\left\{0,1,2\right\}}^{N}$ is a vector of genotype scores at marker m in generation t, ${\hat{\alpha }}_{m}\in \mathbb{R}$ is a marker effect at marker m, and $p_{m}^{\left(t\right)}\in \mathbb{R}$ is a minor allele frequency at marker m in generation t. Here, we considered the following three methods to examine the impact of selection intensity on progeny allocation optimization.

A. SI1: Selected top n=15 genotypes from the current population.

B. SI2: First performed hierarchical clustering based on marker genotypes, creating 25 clusters. Then, it selected only the top one genotype from each cluster, resulting in n=25 genotypes.

C. SI3: First performed hierarchical clustering based on marker genotypes, creating 10 clusters. Then, it selected top five genotypes from each cluster, resulting in n=50 genotypes.

Here, since the selection intensity is formally defined by the selection differential and phenotypic variance, the actual selection intensity at the selection step is determined by both the number of parents selected and how those parents are selected. Therefore, SI1, SI2, and SI3 can indeed be characterized as selection strategies that differ in their selection intensity at the selection step.

For both SI2 and SI3, we performed hierarchical clustering using the genomic relationship matrix calculated from marker genotypes. We computed this matrix using the calcGRM function from the RAINBOWR package with version 0.1.35 (Hamazaki and Iwata 2020) and conducted hierarchical clustering with the hclust function from the stats package with version 4.4.1 (R Core Team 2024).

#### Mating pair candidates for the next generation

Based on the selected n parent candidates, mating pair candidates for the next generation t+1 were determined by a half diallel crossing including selfing, resulting in $K=\frac{n\left(n+1\right)}{2}$ pair candidates in total.

#### Select pairs and allocate progenies to each pair

Next, we employed the following three strategies for the selection of pairs and allocation of progenies. These progeny allocation strategies influence the selection differential in the next generation, thereby affecting the overall selection intensity. Here, the population size was held constant at N=250 individuals throughout the breeding scheme in this study, so the total number of individuals allocated across all mating pairs equals N for all allocation strategies. We set this value at N=250 to represent a small-scale breeding scheme that can be managed by a few breeders, considering phenotyping and cultivation costs. Also, to account for crossing costs, we implemented a constraint requiring that progeny allocations be made in units of at least L=5 individuals per pair.

A. Optimized Resource Allocation (ORA)

The first strategy was the optimized progeny allocation proposed by Hamazaki and Iwata (Hamazaki and Iwata 2024), which directly optimized the number of progenies allocated to each pair candidate, $b_{k}^{\left(t\right)}\in \mathbb{N}$, by integrating future-oriented simulations and the black-box-based optimization technique. Here, ORA refers to the progeny allocation strategy optimized by the AI breeder. Inside the breeding simulations by the AI breeder for the allocation optimization, the current generation was initialized as $\tau =\tau_{0}$, where $\tau_{0}\in \left\{0,\;t_{u}\right\}$ represented an initial generation number in breeding simulations in the scheme with model updates (Supplemental Fig. 2). In other words, the AI breeder optimized allocation strategies at t=0 and $t=t_{u}$, coinciding with the GP model updates (Supplemental Fig. 1). The final generation in the breeding simulations was set as $T_{s}\in \left\{2,3\right\}$ for $\tau_{0}=0$ and $T_{s}=4$ for $\tau_{0}=t_{u}$. This means that during the initial optimization process, we prepared two variants of the ORA depending on the target generation (ORA2 for $T_{s}=2$ and ORA3 for $T_{s}=3$), whereas during the second optimization of progeny allocation, we consistently targeted the final generation in the scheme intended for practical breeding ($T_{s}=T_{r}=4$). Here, no model updates were implemented inside the breeding simulations performed by the AI breeder (Supplemental Fig. 2). Details of the ORA algorithm are given in the later subsection of *Optimization of progeny allocation strategies*.

B. EQual allocation (EQ)

The second strategy was equal allocation, which distributed progeny evenly among mating pairs. When the number of potential mating pair candidates K exceeded the population size N, we randomly selected $K^{\prime }=\frac{N}{L}=$50 pairs from all mating pair candidates and allocated $b_{k}^{\left(t\right)}=L$ progenies to each selected pair.

C. Optimal Cross Selection (OCS)

The third strategy was the OCS approach, which selected crossing pairs by considering both the GEBVs of the selected individuals and their genetic diversity (Allier *et al.* 2019b, Gorjanc *et al.* 2018). Details are provided in Supplemental Text 1, but we implemented OCS following the method in Sakurai *et al.* (Sakurai *et al.* 2024), which resulted in the selection of $K^{\prime \prime }=25$ pairs from all mating pair candidates. After selecting these $K^{\prime \prime }$ pairs, $b_{k}^{\left(t\right)}=10$ progenies were allocated to each selected mating pair.

#### Generate progeny for the next generation

From mating pair k, $b_{k}$ progenies were produced following the principle of meiosis, resulting in a new population with generation t+1 consisting of N progenies. Here, recombination rates used in the meiosis simulation were calculated using the Kosambi map function (Kosambi 1943, Zhao and Speed 1996) applied to the linkage map generated by GENOME.

#### Repeat the selection and mating processes

Steps 2–6 were repeated until reaching the population with the final generation $T_{r}=4$, assuming a scenario where rapid genetic improvement was needed in small-scale breeding schemes.

### Evaluation of the genetic gains of breeding schemes

We evaluated the results for each strategy using breeding values at generation t, i.e., ${𝐮}^{\left(t\right)}={𝐖}^{\left(t\right)}𝛂$, particularly for the final population with generation $T_{r}$. Here, to evaluate the population’s maximum under the breeding strategy, we computed ${\tilde{u}}^{\left(\text{top}\right)\left(t\right)}$, the empirical mean of ${𝐮}^{\left(t\right)}$ for the top five genotypes. These genotypes were selected based on their ${𝐮}^{\left(t\right)}$ values in descending order. We then scaled ${\tilde{u}}^{\left(\text{top}\right)\left(t\right)}$ by the initial population’s mean and standard deviation, $u^{\left(\text{mean}\right)\left(0\right)}$ and $\sigma_{u}^{\left(0\right)}$, resulting in $u^{\left(\text{top}\right)\left(t\right)}=\frac{{\tilde{u}}^{\left(\text{top}\right)\left(t\right)}-u^{\left(\text{mean}\right)\left(0\right)}}{\sigma_{u}^{\left(0\right)}}$. We selected the top five genotypes for evaluation to represent potential for new variety development while mitigating stochastic variation between simulations. For each parent panel simulation dataset, we ran 10,000 breeding schemes per strategy and calculated the genetic gain for evaluation as the empirical mean of $g^{\left(t\right)}=u^{\left(\text{top}\right)\left(t\right)}/u^{\left(\text{top}\right)\left(0\right)}$ from these results. We repeated the simulation and evaluation processes for 10 replicates of the phenotype simulation, each with different QTL positions and effects.

### Optimization of progeny allocation strategies

In this study, the number of progenies allocated to pair k at generation τ, $b_{k}^{\left(\tau \right)}$, was determined by applying the softmax function to the weighted sum of the multiple “goodness” metrics (Supplemental Fig. 3), following Hamazaki and Iwata (2024). Since we need to incorporate the constraint on the minimum allocation units L, $b_{k}^{\left(\tau \right)}$ was computed as shown in Equation 5.


$$b_{k}^{\left(\tau \right)}=\left\lfloor \frac{N}{L}\;\times \;\frac{\exp\left({{𝛚}_{k}^{\left(\tau \right)}}^{⊤}\;{𝛉}^{\left(\tau \right)}\right)\;}{\sum_{k}\exp\left({{𝛚}_{k}^{\left(\tau \right)}}^{⊤}\;{𝛉}^{\left(\tau \right)}\right)}\right\rfloor \times L,$$ (5)

where ${𝛚}_{k}^{\left(\tau \right)}\in {\mathbb{R}}^{d}$ is a feature vector of goodness metrics, ${𝛉}^{\left(\tau \right)}\in {\mathbb{R}}^{d}$ is a vector that determines the weight given to each goodness metric, thus becoming a parameter to be optimized by the AI breeder, and ⌊⋅⌋ is the floor function. Here, as in Equations 1 and 2, the breeding scheme can be viewed as a black-box function F with ${\left\{{𝛉}^{\left(\tau \right)}\right\}}_{\tau =\tau_{0},\ldots ,T_{s}-1}$ as input and the final genetic gain, $g^{\left(T_{s}\right)}=u^{\left(\text{top}\right)\left(T_{s}\right)}/u^{\left(\text{top}\right)\left(\tau_{0}\right)}$, as output. Thus, we employed a black-box optimization technique called StoSOO (Valko *et al.* 2013) to estimate the weighting parameters that maximized ${𝔼}_{𝛈}\left[g^{\left(T_{s}\right)}\right]$, following Hamazaki and Iwata (2024). In this study, we conducted 50 breeding simulations to calculate the expectation with respect to 𝛈 for one function evaluation (Supplemental Fig. 2) and performed 10,000 or 20,000 function evaluations to obtain the optimal set of weighting parameters for breeding schemes with or without model updates, respectively (Fig. 1A, 1B). Then, the optimized $\left\{{\hat{𝛉}}^{\left(\tau \right)}\right\}$ was utilized for progeny allocation in the real breeding schemes conducted by actual breeders.

As candidates for the feature vector of goodness metrics ${𝛚}_{k}^{\left(\tau \right)}$, we used the WBV (Goddard 2009, Jannink 2010) and the expected genetic variance of progeny (GVP) for each mating pair (d=2). The first metric, WBV, for pair k was calculated as the mean of the two parents’ WBVs in Equation 4, i.e., $\omega_{k1}^{\left(\tau \right)}=\frac{1}{2}\sum_{i\in \left\{\text{MAT},\;\text{PAT}\right\}}u_{k_{i}}^{\left(\text{WBV}\right)\left(\tau \right)}$, where $k_{\text{MAT}}$ and $k_{\text{PAT}}$ correspond to the indices of the parents. The second metric, GVP, was assessed as the genetic diversity of gametes produced after self-fertilization of progeny from each mating pair. The GVP for mating pair k can be theoretically derived using approaches proposed by previous studies (Allier *et al.* 2019a, 2019b, Lehermeier *et al.* 2017), as shown in the following Equation 6.


$$\omega_{k2}^{\left(\tau \right)}=\sum_{m=1}^{M}\sum_{m^{\prime }=1}^{M}\sigma_{kmm^{\prime }}^{\left(\tau \right)}{\hat{\alpha }}_{m}{\hat{\alpha }}_{m^{\prime }}\;,$$ (6)

where $\sigma_{kmm^{\prime }}^{\left(\tau \right)}$ represents the covariance between markers m and $m^{\prime }$ resulting from progeny segregation derived from pair k at generation τ. The detailed derivation of $\sigma_{kmm^{\prime }}^{\left(\tau \right)}$ is provided in Allier *et al.* (2019b), but $\sigma_{kmm^{\prime }}^{\left(\tau \right)}$ can be computed as a function of allelic scores of the parents’ haplotypes and the recombination rates between markers. Here, when markers m and $m^{\prime }$ are located on different chromosomes, $\sigma_{kmm^{\prime }}^{\left(\tau \right)}=0$, and this term does not contribute to the GVP.

## Results

### Change in the genetic gains over four generations

We compared three allocation strategies (ORA, EQ, and OCS) in breeding schemes without model updates, and four strategies (ORA2, ORA3, EQ, and OCS) in schemes with model updates. First, we plotted the genetic gains over four generations across two scenarios for the number of QTLs (Scenario 1 and Scenario 2) under three selection intensities (SI1, SI2, and SI3) (Fig. 3, Supplemental Fig. 4).

In the breeding schemes without model updates, genetic gains reached saturation at generation t=2 for all allocation strategies across both scenarios and all selection intensities (Supplemental Fig. 4). In Scenario 1 with a small number of QTLs, comparative analysis of allocation strategies revealed that ORA exhibited superior performance over both OCS and EQ under high selection intensity (SI1), whereas under lower selection intensities (SI2, SI3), the genetic gains were maximized by OCS, followed by ORA and EQ (Supplemental Fig. 4A). Conversely, in Scenario 2 with a large number of QTLs, ORA demonstrated genetic gains that were either equal to or greater than OCS across all selection intensities, while consistently outperforming EQ (Supplemental Fig. 4B). Given that genetic gains quickly plateaued across all allocation strategies, implementing model updates are necessary for developing more accurate breeding strategies.

When examining breeding schemes with model updates, genetic gains continued to improve after the model update at generation t=2, progressing until the final generation (t=4) across all scenarios, selection intensities, and allocation strategies (Fig. 3). This trend was particularly pronounced in Scenario 2, suggesting that model updates are highly effective for quantitative traits governed by a large number of QTLs (Fig. 3B). In Scenario 1 with fewer QTLs (Fig. 3A), ORA strategies performed equally or worse compared to EQ and OCS under high selection intensity (SI1), yet exhibited superior genetic gains under lower selection intensities (SI2, SI3). A similar trend was observed in Scenario 2 with numerous QTLs (Fig. 3B); however, there were two notable differences from Scenario 1: ORA3 demonstrated superior performance compared to other allocation strategies even under SI1, and OCS clearly outperformed EQ under SI2 and SI3, although it still could not match ORA’s performance. When comparing the two ORA strategies, ORA3 consistently outperformed ORA2 except under SI3 conditions in Scenario 2, though the performance difference was modest (Fig. 3).

### Genetic gains across different simulation repetitions

To evaluate the characteristics of each allocation strategy comprehensively, we analyzed the cumulative distribution functions (CDFs) of genetic gain in the final generation (t=4) across two scenarios under three selection intensities, using 10,000 simulation repetitions. Fig. 4 and Supplemental Fig. 5 show these results, with the horizontal axis indicating final genetic gain and the vertical axis indicating the percentile of simulation repetitions. The 1st and 99th percentiles represent the worst and best performances among the 10,000 repetitions. Effectiveness of each strategy is indicated by how far its CDF curve shifts rightward and downward—the more significant this shift, the better the overall performance of the strategy.

Regarding the comparison between allocation strategies, the CDF curves showed similar trends to those observed in Fig. 3 and Supplemental Fig. 4 (Fig. 4, Supplemental Fig. 5), with the ranking of strategies based on the CDF curves corresponding well to the ranking observed for final genetic gain in Fig. 3 and Supplemental Fig. 4. For both schemes with and without model updates, the CDF curves for the ORA strategies in Scenario 2 (Fig. 4B, Supplemental Fig. 5B) showed smoother and gentler slopes than those in Scenario 1 (Fig. 4A, Supplemental Fig. 5A), suggesting that the optimization for traits governed by numerous QTLs led to greater variations among simulation repetitions. In Scenario 1 with model updates, the CDF curves exhibited slopes almost parallel to the vertical axis for all allocation strategies under high selection intensity, and for the optimized allocation strategies under low selection intensities (Fig. 4A). These results indicated that applying strong selection intensity to traits controlled by a small number of QTLs leads to consistent outcomes across different simulation runs. In addition, in Scenario 2 with model updates, while the ORA strategies performed comparably to OCS in the best-performing case, it consistently outperformed OCS in the worst-performing case, indicating that they can provide more stable results for traits controlled by numerous QTLs (Fig. 4B).

### Genetic gains across different phenotype simulations

We further evaluated genetic gains in the final generation (t=4) using ten replications of phenotype simulations to verify that the ORA strategies outperformed the EQ and OCS strategies across traits with varying QTL positions, effects, and errors. For these simulations, we changed QTL positions and effects while maintaining consistent QTL numbers and effect distributions. To reduce computational time, we determined the ORA strategies after 2,500 function evaluations for schemes with model updates and 5,000 for schemes without. The expected genetic gains for all ten replications appear in Supplemental Figs. 6 and 7, while the improvement rates compared to EQ, calculated by subtracting the genetic gains of EQ from those of ORA or OCS and then dividing by the genetic gains of EQ, are presented in Fig. 5 and Supplemental Fig. 8.

While the ORA strategies were unable to outperform EQ under high selection intensity, they were markedly superior to EQ under low selection intensities (Fig. 5, Supplemental Figs. 6–8). This trend was particularly pronounced when model updates were implemented in Scenario 2 with a large number of QTLs (Fig. 5B, Supplemental Fig. 6B). In addition, comparing the ORA strategies with OCS revealed that in Scenario 2, ORAs failed to match or exceed OCS, regardless of whether model updates were implemented (Fig. 5B, Supplemental Fig. 8B). However, in Scenario 1, ORA strategies surpassed OCS in improvement rates (Fig. 5A, Supplemental Fig. 8A). These findings suggest that the ORA strategies are particularly effective for traits controlled by a small number of QTLs. Furthermore, in Scenario 2, where traits are governed by numerous QTLs, OCS exhibited greater variability across different phenotype simulations, indicating that ORA might be more effective in developing varieties with consistent genetic gains across diverse traits (Fig. 5B, Supplemental Fig. 8B).

### Change in the genetic variances over four generations

We then compared genetic diversity between ORA, EQ, and OCS strategies across four generations, examining schemes both with (Supplemental Fig. 9) and without model updates (Supplemental Fig. 10) under three selection intensities for Scenario 1 and Scenario 2. Genetic diversity within each generation’s breeding population was measured using the genetic variance of true genotypic values, i.e., $\sigma_{u}^{{\left(t\right)}^{2}}$.

In Scenario 1, genetic variance increased compared to the initial population until generation t=2, regardless of whether model updates were implemented (Supplemental Figs. 9A, 10A). Subsequently, breeding populations without model updates maintained stable genetic variance (Supplemental Fig. 10A). In contrast, when model updates were performed, genetic variance declined for all strategies under SI1, and for all strategies except EQ under SI2 and SI3 (Supplemental Fig. 9A). In Scenario 2, both ORA and EQ strategies showed a slight increase in genetic variance in the final generation compared to the initial generation, regardless of whether model updates were performed, while OCS exhibited a gradual decrease (Supplemental Figs. 9B, 10B). When comparing allocation strategies in the final generation, ORA maintained lower genetic variance than EQ (except in Scenario 1 without model updates), but preserved higher genetic variance than OCS.

### Change in the prediction accuracies over four generations

Finally, we assessed prediction accuracy across four generations by calculating the correlation between true and estimated genotypic values in each generation for Scenario 1 and Scenario 2 for the scheme with (Fig. 6) and without model updates (Supplemental Fig. 11).

First, without model updates, prediction accuracy declined to nearly zero by the final generation (t=4) in both scenarios across all selection intensities and allocation strategies (Supplemental Fig. 11). Most notably, except for EQ under SI2, accuracy had already fallen to approximately 0.2 by t=2, indicating rapid population changes from selection and mating.

In contrast, implementing model updates at t=2 restored prediction accuracy to approximately 0.75 across all scenarios, selection intensities, and allocation strategies—comparable to those in the initial population (Fig. 6). Although accuracy gradually decreased toward the final generation, breeding schemes with model updates maintained substantially better accuracy than those without updates.

Between scenarios, Scenario 2, with more QTLs controlling traits, showed faster decreases in prediction accuracy than Scenario 1 (Fig. 6B and Supplemental Fig. 11B compared with Fig. 6A and Supplemental Fig. 11A), though model updates effectively restored accuracy to similar levels in both scenarios. Among allocation strategies, OCS consistently showed the lowest prediction accuracy (Fig. 6, Supplemental Fig. 11), particularly in Scenario 2 where accuracy plummeted to approximately 0.05 by generation t=1. For ORA and EQ strategies, EQ showed faster decline in accuracy under high selection intensity (SI1), while ORA variants declined faster under moderate and low selection intensities (SI2 and SI3). Interestingly, in Scenario 2, EQ showed marked accuracy decreases under high selection intensity, whereas ORA exhibited similar pattern of accuracy decline across all selection intensities (Fig. 6B, Supplemental Fig. 11B). Between ORA variants, ORA3 showed slightly greater reduction in accuracy than ORA2, though differences were minimal.

## Discussion

Breeding schemes involve multiple decision-making points, such as selection and mating. Recently, numerous breeding strategies leveraging genomic information have emerged (Diot and Iwata 2022, Hamazaki and Iwata 2024, Moeinizade *et al.* 2019, Sakurai *et al.* 2024, Wang *et al.* 2018). Previous research, including work by Hamazaki and Iwata (2024), often developed and optimized breeding strategies assuming that true QTL effects are known (Amini *et al.* 2021, Daetwyler *et al.* 2015, Goiffon *et al.* 2017, Hamazaki and Iwata 2024, Hamazaki *et al.* 2025, Kemper *et al.* 2012, Moeinizade *et al.* 2019, 2022, Wang *et al.* 2018, Zhang and Wang 2022). However, in practical breeding schemes, true QTL effects are seldom fully understood, requiring the use of marker effects estimated by GP instead. Thus, in this study, we optimized allocation strategies using estimated marker effects while incorporating model updates, and subsequently evaluated their effectiveness. The following discussion examines our findings on genetic gains and genetic variances, and assesses whether optimized allocation strategies remained effective when using estimated marker effects rather than true QTL effects.

### Discussion on genetic gains

First, examining the changes in genetic gains revealed that while genetic gain plateaued without model updates (Supplemental Fig. 4), implementing model updates consistently improved gains through the final generation (Fig. 3). This improvement is strongly linked to enhanced prediction accuracy from these updates (Fig. 6), as making selection, mating, and allocation decisions using more accurate models allowed genetic gain to increase through the final generation. Notably, the improvement between generations t=2 and t=3 exceeded that between t=1 and t=2, likely because the prediction accuracy recovered after model updates at t=2, enabling more effective selection.

Our analysis revealed interesting patterns in the relationship between selection intensity and allocation strategy (Figs. 3–5, Supplemental Figs. 4–8). When selection intensity was high (SI1), the optimized allocation strategy (ORA) achieved genetic gains similar to or lower than those of equal allocation (EQ). However, under lower selection intensity conditions (SI2, SI3), ORA significantly outperformed EQ. This finding supports the conclusion by Hamazaki and Iwata (2024) that optimizing allocation strategy becomes more crucial when selection intensity is low.

Additionally, under low selection intensity conditions, model updates enabled ORA to achieve higher genetic gain than both EQ and OCS in both scenarios (Figs. 3, 4, Supplemental Figs. 4, 5). This demonstrates that ORA functions effectively with model updates because allocation strategies are re-optimized simultaneously with the models. When comparing ORA2 and ORA3 with 10,000 function evaluations, ORA3 generally produced superior results except for SI3 under Scenario 2, suggesting that optimization strategies focused on generations beyond the next model update are usually more effective than those targeting only the immediate next model update (Figs. 3, 4). Consistent with this observation, ORA3, with its long-term approach, often demonstrated a modest advantage in maintaining genetic variance compared to ORA2 (Supplemental Fig. 9), particularly in Scenario 1 where traits are controlled by a small number of QTLs, showing a more gradual decline even when genetic variance decreased rapidly after generation 2.

### Discussion on genetic variances

In this study, genetic variances tented to increase in the first two generations for all allocation strategies (Supplemental Figs. 9, 10). While selection would generally be expected to reduce genetic diversity, this counterintuitive result stems from the experimental conditions in this study. In this study, the initial breeding population was assumed to be close to germplasm collections, so rare alleles might also be assigned genetic effects. Therefore, in the initial stages, these rare alleles were favored, shifting allele frequencies toward 0.5 and thereby contributing to the increase in genetic variances—a phenomenon observed in other studies as well (Jannink 2010). It should be noted that in simulations using populations derived from bi-parental or four-way crosses as initial populations, as in Sakurai *et al.* (2024), allele frequencies generally start close to 0.5 rather than 0, preventing such increases from occurring.

With model updates implemented, subsequent generations showed different patterns in genetic variances. In Scenario 1 with fewer QTLs, genetic variances tended to decrease as alleles became fixed and minor allele frequencies approached 0 (Supplemental Fig. 9A). In contrast, Scenario 2 with more QTLs showed smaller decreases in genetic variances because many alleles remained that were not moving toward fixation (Supplemental Fig. 9B). Although our simulations spanning four generations did not reveal large decreases in genetic variance, we expect genetic variances to decrease rapidly in both scenarios during longer-term breeding schemes. In simulations without model updates, prediction accuracies in the later stages of breeding schemes were inherently low, causing individuals to be selected more randomly, which tended to suppress the decrease in genetic variances (Supplemental Fig. 10).

### Comparison of ORA and OCS

From the results of different simulation repetitions, while ORA and OCS showed similar performance in the best-performing cases, ORA significantly outperformed OCS in the worst cases (Fig. 4, Supplemental Fig. 5). This indicates that ORA can consistently deliver high-quality varieties even when random factors such as recombination act unfavorably.

Moreover, from the results of different phenotype simulations, particularly in Scenario 1 assuming traits controlled by a small number of QTLs, ORA variants showed better genetic gains than OCS at lower selection intensities (Fig. 5A, Supplemental Figs. 6A–8A). In contrast, for Scenario 2 assuming traits controlled by many QTLs, ORA showed no clear advantage over OCS (Fig. 5B, Supplemental Figs. 6B–8B). However, ORA showed less variation in results, suggesting it may have stability that can accommodate various traits. Additionally, since this study terminated the black-box optimization for ORA prematurely due to limitations in computational time, it is possible that spending more time on optimization could surpass OCS. Indeed, focusing on the results for one phenotype simulation confirmed the advantage of ORA in both scenarios (Figs. 3, 4, Supplemental Figs. 4, 5).

Furthermore, while OCS achieved high genetic gains through strong selection pressure, evidenced by the sharp decrease in prediction accuracy from t=0 to t=1, ORA achieved comparable genetic gains while better controlling rapid changes in population structure than OCS (Fig. 6, Supplemental Fig. 11). As confirmed when updating models in Scenario 2, ORA variants effectively balanced the overall selection intensity across both selection and allocation stages through its optimized allocation strategy, shown by consistent prediction accuracy changes regardless of selection intensity (Fig. 6B). Although this study focused on short-term breeding schemes and did not observe significant decreases in genetic variances, in longer-term breeding schemes, this appropriate adjustment of selection intensity is expected to contribute to improving genetic gain while maintaining genetic diversity.

Based on these results, ORA is considered to be a robust approach that not only demonstrates excellent performance but also maintains stability when faced with diverse target traits or random factors such as recombination.

### Future prospects and conclusions

Our study demonstrates that the optimized allocation strategies, when combined with appropriate model updates, deliver excellent performance and robustness in realistic breeding schemes that rely on estimated marker effects. By conducting simulations under more realistic assumptions, our study bridges the gap between theoretical approaches and practical breeding applications, making the optimization framework valuable for plant breeders in their daily decision-making. We believe this methodology can be readily implemented by breeding programs worldwide to enhance genetic gain while maintaining diversity.

However, we found that these strategies become less effective under high selection intensity. In future works, we plan to address this limitation by optimizing selection intensity and allocation strategy simultaneously. Additionally, since model updates have enabled longer-term breeding programs, future challenges include performance verification in long-term breeding schemes and application of the optimized allocation strategies in real-world breeding schemes with field trials. Furthermore, when considering long-term breeding programs, simultaneous improvement of multiple traits becomes increasingly important, especially in major crops. Therefore, optimizing breeding strategies that account for trade-offs among multiple traits represents a critical theme for future research.

We have emphasized that the optimization of progeny allocation strategies proposed in this study is particularly effective for small-scale breeding schemes, as the benefits of optimal resource allocation are expected to be greater under more limited resource constraints. However, the same framework can certainly be applied to larger-scale breeding programs, where optimizing allocation strategies would remain highly significant. In particular, when model updates are performed, larger population sizes are expected to improve prediction accuracy, enabling sustained high-precision optimization over longer periods. Furthermore, in this study, we focused on cross-pollinated and vegetatively propagated crops because genotypes are not fixed in the final generation. However, by modifying the breeding simulation to include fixation and determining allocation strategies that maximize genetic gains in the fixed generation, the framework can be extended to self-pollinated crops as well. In particular, by adopting a two-part strategy consisting of population improvement and product development (Gaynor *et al.* 2017, Gorjanc *et al.* 2018), the challenge of lengthy model update times in self-pollinated crops can be largely addressed. This approach enables model updates for recurrent selection to occur simultaneously with fixation, allowing allocation strategies to be optimized effectively under realistic model update frequencies. Thus, our framework for breeding strategy optimization with model updates proposed in this study is highly extensible with great potential for future development.

Furthermore, we aim to develop more efficient methods for optimizing breeding decisions by leveraging advanced technologies like automatic differentiation as proposed in Hamazaki *et al.* (2025). Through these efforts, the integration of breeding simulations and optimization approaches as proposed by Hamazaki and Iwata (2024), extended in our study via updates of the GP models and the optimal allocation strategies, will bring significant innovation in genome-assisted breeding.

## Author Contribution Statement

KH, HI, and KT designed the study; KH implemented all the resource allocation strategies; KH performed the simulation study; KH interpreted results and drafted the manuscript; KT supervised the study. All authors read and approved the final manuscript.

## Supplementary Material

Supplemental Figures

Supplemental Table

Supplemental Text

## Figures and Tables

**Fig. 1.**
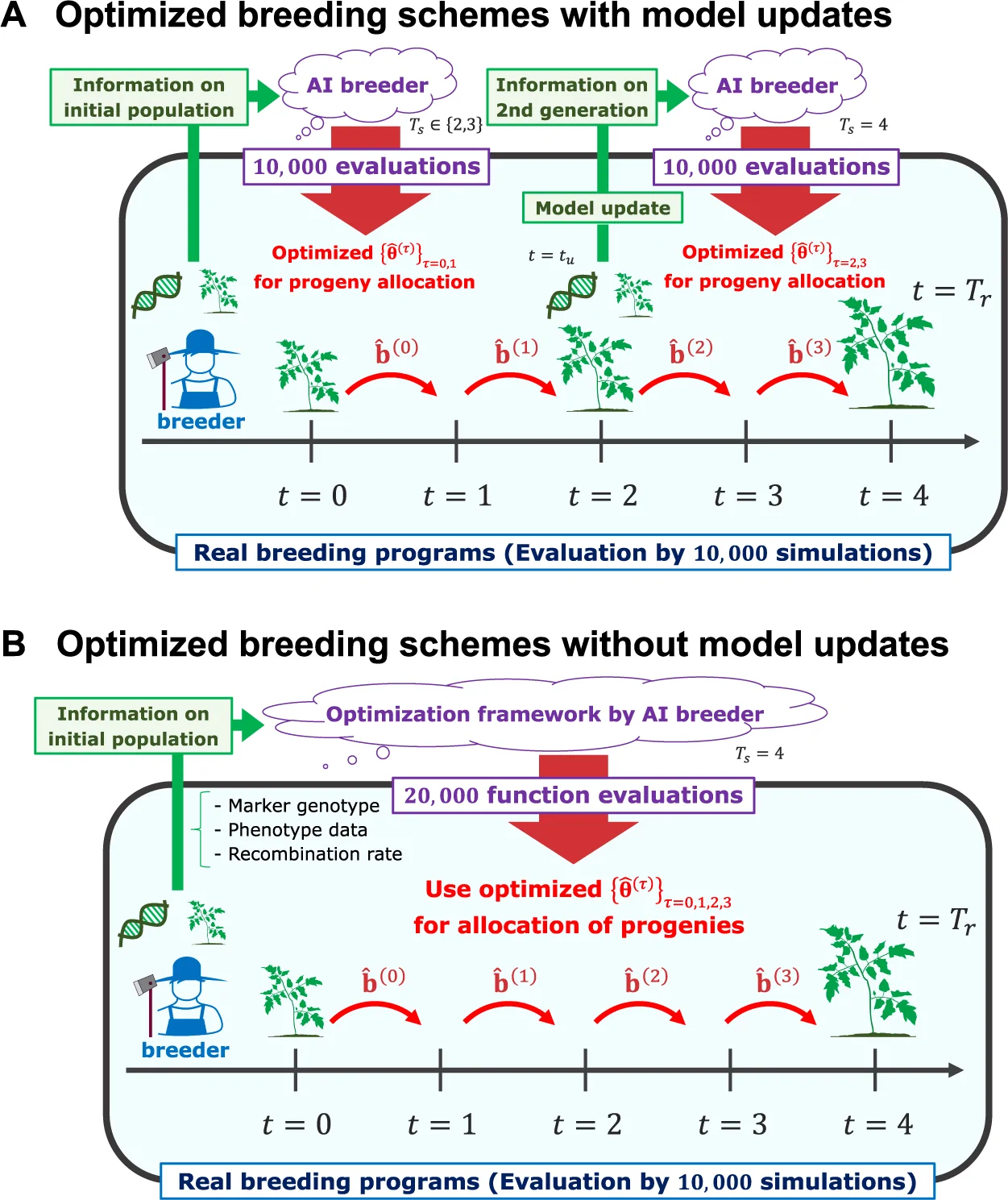
Optimization of allocation strategies in breeding schemes with or without model updates and their implementation intended for practical breeding programs. Panel (A) corresponds to the scheme with model updates, while panel (B) corresponds to the scheme without model updates. In the panel (A), we not only estimate marker effects and optimize allocation strategies in the initial population, but also examine updating the GP model and re-optimizing allocation strategies at an intermediate stage (t=2) as the breeding scheme progresses under the optimized allocation strategy. Panel (B) is adapted from the Figure 1 in (Hamazaki and Iwata 2024).

**Fig. 2.**
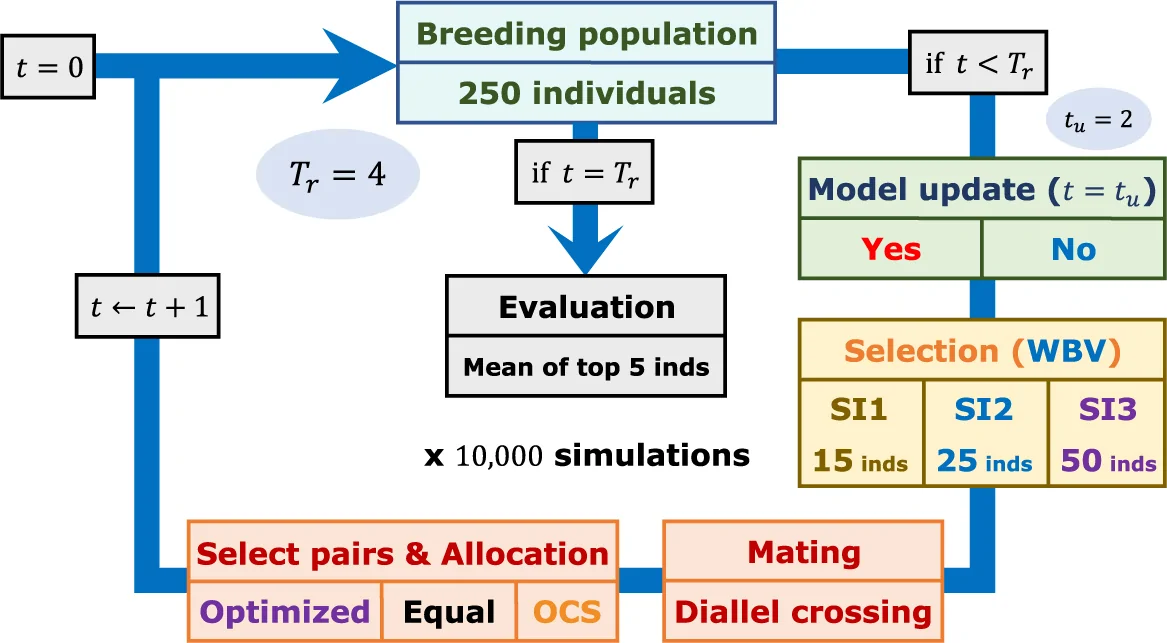
Images of breeding schemes intended for practical breeding assumed in this study. Similar to (Hamazaki and Iwata 2024), we assumed small-scale plant breeding programs with recurrent selection. In a scheme, the selection, mating, and allocation steps were repeated until the final generation. Here, we prepared for the two scenarios with different numbers of QTLs and three selection strategies with different selection intensities.

**Fig. 3.**
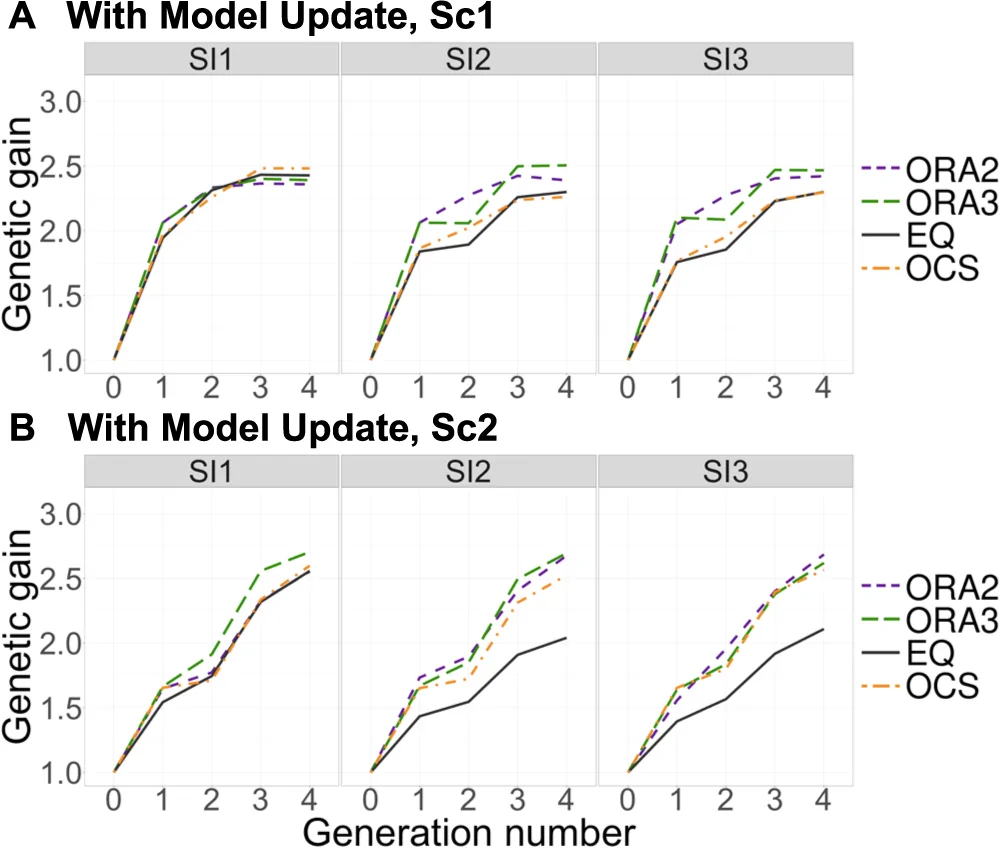
Change in the expected genetic gains over four generations under different selection intensities for the scheme with model updates. The horizontal and vertical axes represent the number of generations and the genetic gains, respectively. We compared the following allocation strategies under the different selection intensities (SI1–SI3): ORA: optimized resource allocation (purple dashed), ORA2: optimized resource allocation with targeting $T_{s}=2$ in the initial optimization (purple dashed), ORA3: optimized resource allocation with targeting $T_{s}=3$ in the initial optimization (green long dotted), EQ: equal allocation (black solid). OCS: optimal cross selection (orange dash-dotted line). Panel (A) corresponds to Scenario 1, while panel (B) corresponds to Scenario 2.

**Fig. 4.**
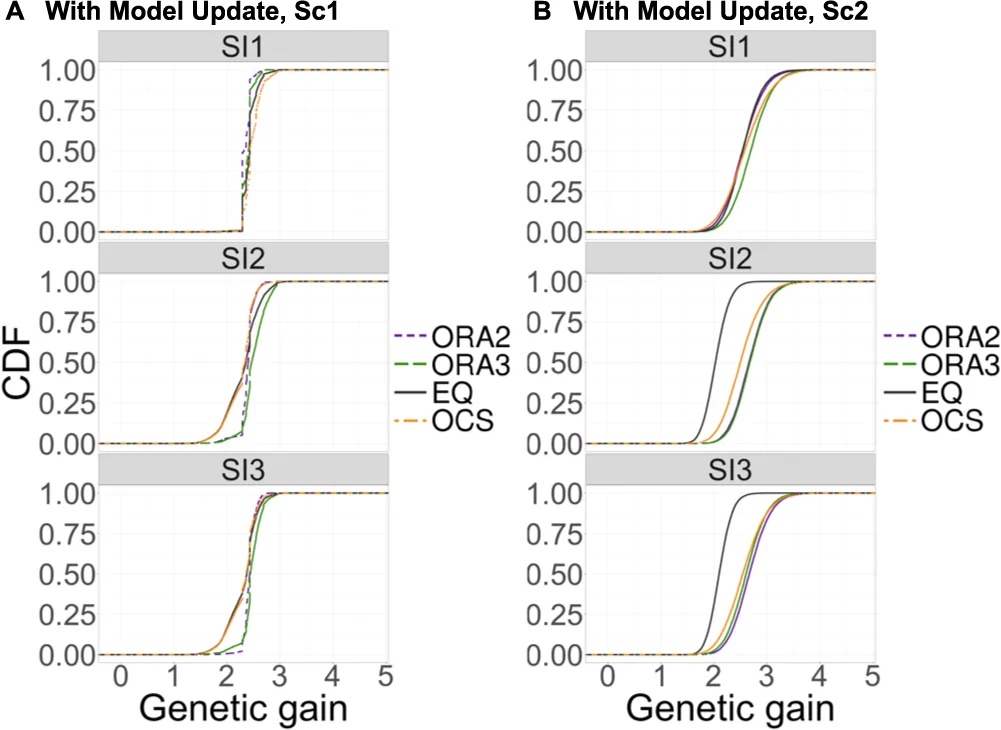
Genetic gains across different simulation repetitions for the scheme with model updates. The horizontal axis shows the final genetic gain of an individual, whereas the vertical axis represents the percentile of the simulation repetitions. Panel (A) corresponds to Scenario 1, while panel (B) corresponds to Scenario 2. The abbreviations of the allocation strategies are the same as those of Fig. 3.

**Fig. 5.**
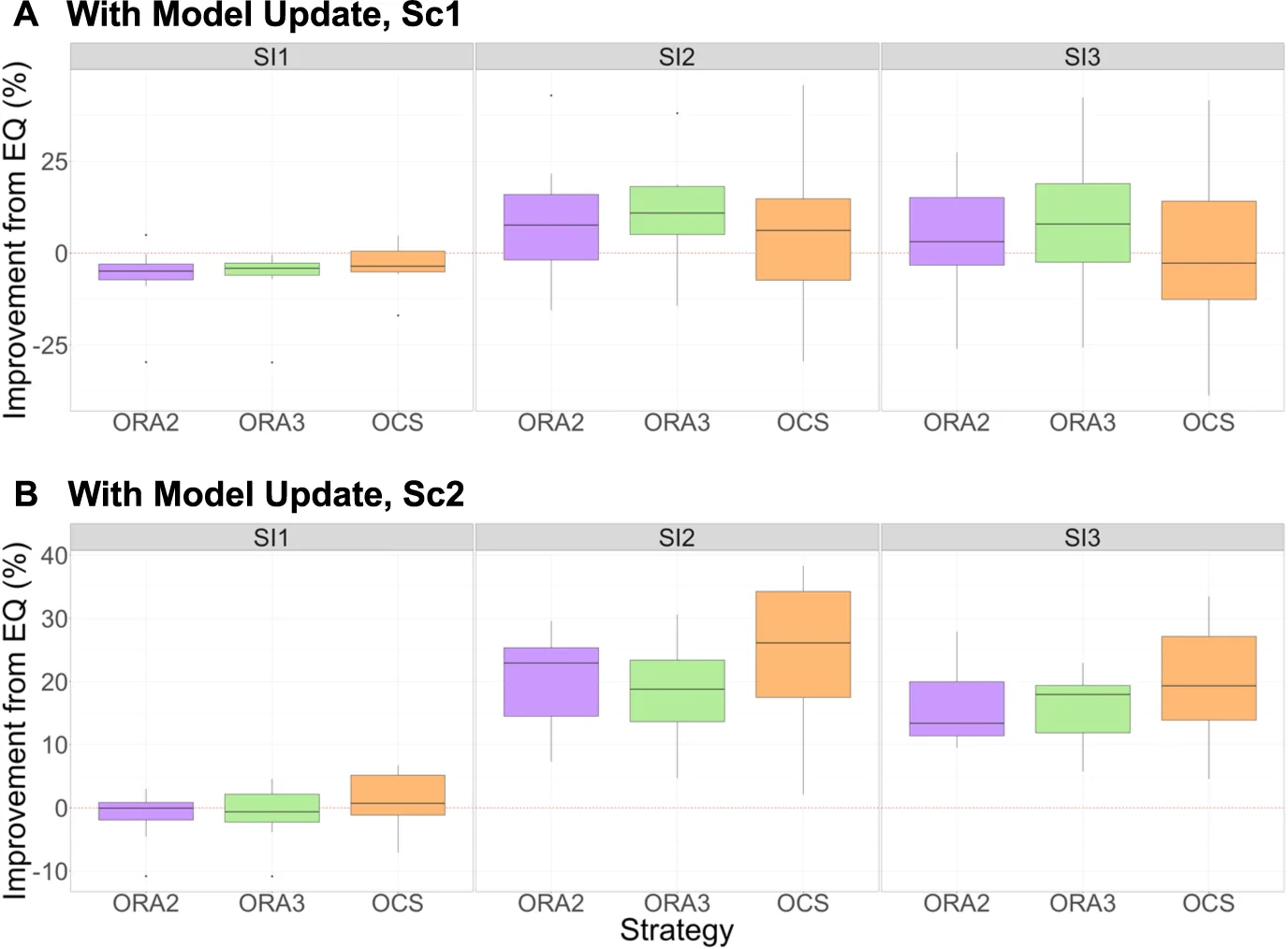
Improvement of the final genetic gains compared to the equal allocation strategy using ten replications for the phenotype simulation under different selection intensities for the scheme with model updates. This figure includes boxplots of the improvement of the final genetic gains compared to the equal allocation strategy using ten replications for the phenotype simulation. The horizontal and vertical axes represent the different allocation strategies and the improvement rate of the final genetic gains, respectively. We compared the following allocation strategies under the different selection intensities (SI1–SI3): ORA (purple), ORA2 (purple), ORA3 (light green), and OCS (orange), with details given in Fig. 3. Panel (A) corresponds to Scenario 1, while panel (B) corresponds to Scenario 2.

**Fig. 6.**
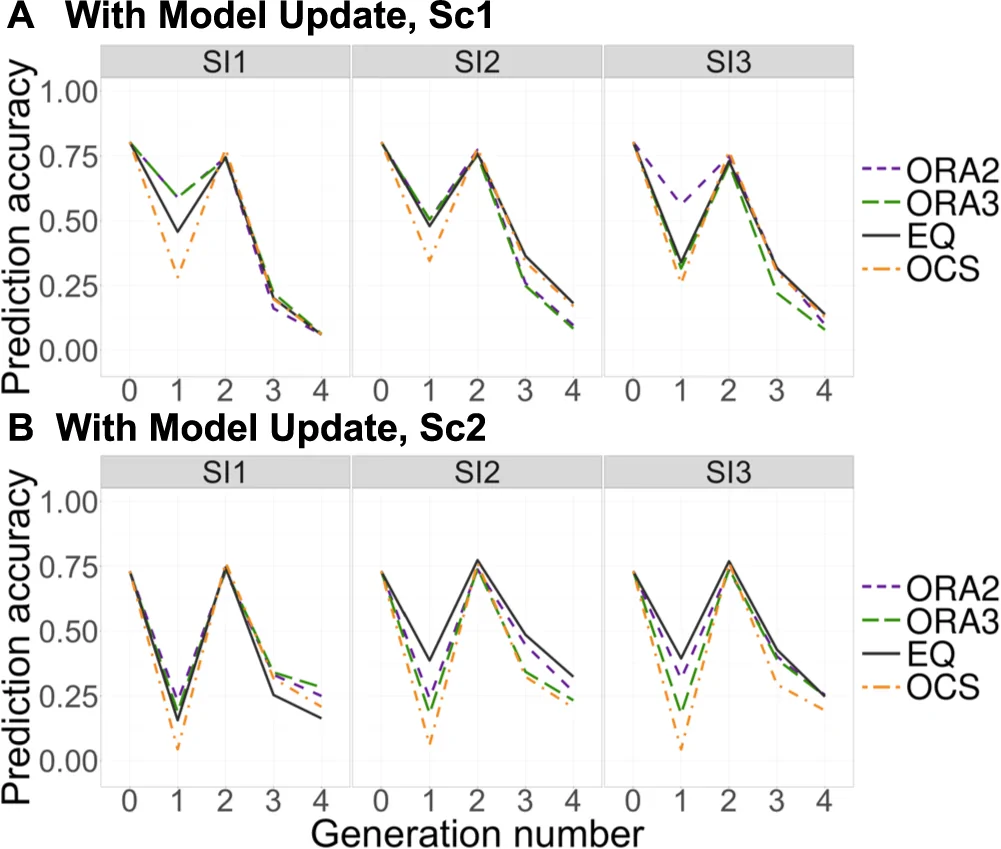
Change in the prediction accuracies over four generations under different selection intensities for the scheme with model updates. The horizontal and vertical axes represent the number of generations and the prediction accuracies for the breeding populations, respectively. Panel (A) corresponds to Scenario 1, while panel (B) corresponds to Scenario 2. The abbreviations of the allocation strategies are the same as those of Fig. 3.
