# Supplementary material for: Optimizing progeny allocation strategies in breeding schemes while updating genomic prediction models
Source: Breed Sci. 2026 Jun 25;76(3):285–98. doi: 10.1270/jsbbs.25056 (PMC13709625; doi:10.1270/jsbbs.25056)
Supplement: Supplementary file 1 [file 76_285_s1.pdf]

doi: 10.1270/jsbbs.25056

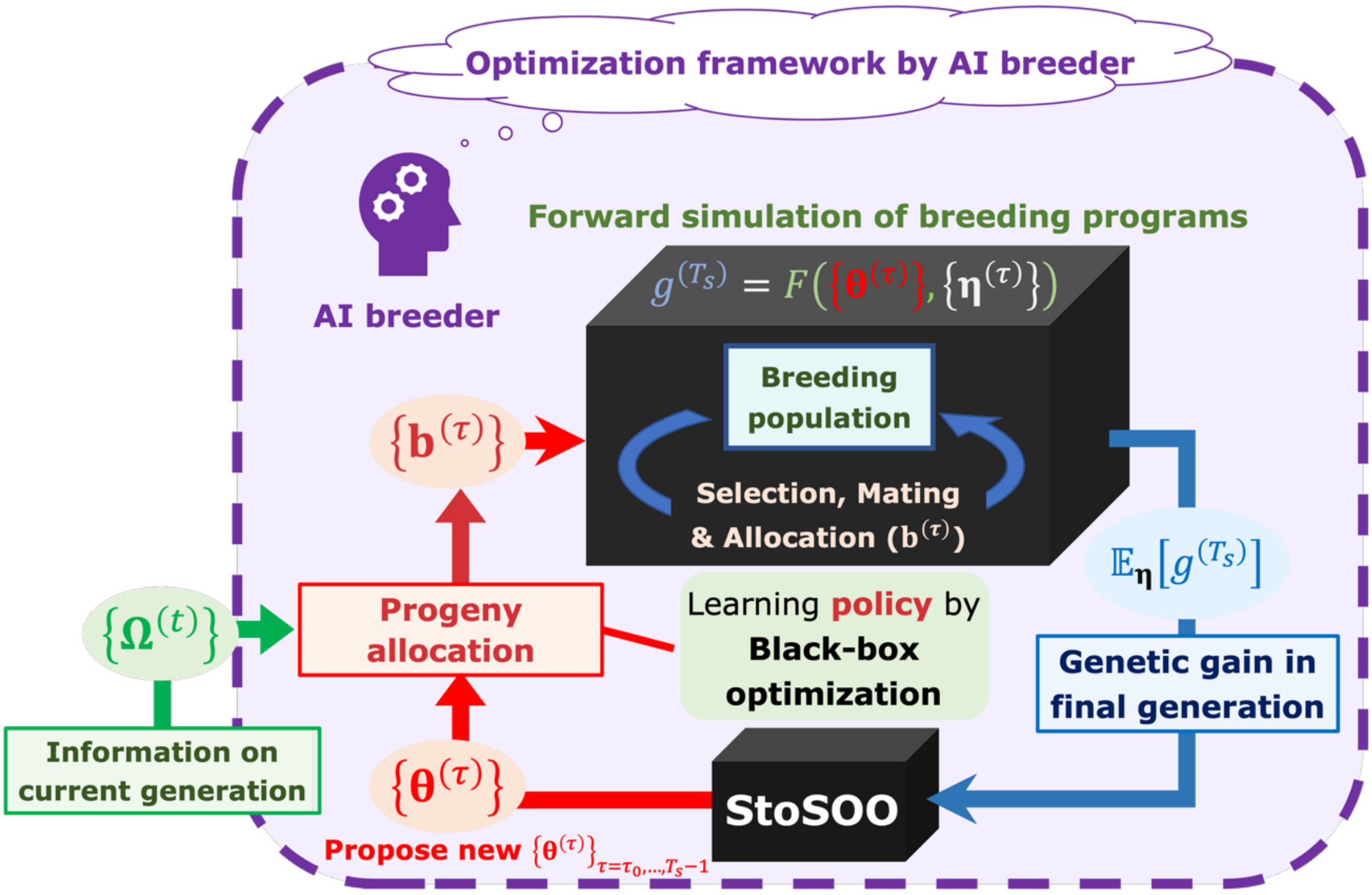

Supplemental Fig. 1. Image of the framework to optimize progeny allocation for each mating pair in the breeding scheme. In our framework, the breeders can obtain a set of parameters representing the optimal allocation strategy, $\{\hat{\theta}^{(\tau)}\}_{\tau=\tau_0,\ldots,T-1}$, by providing the AI breeder with information on an initial breeding population, as in Hamazaki and Iwata (2024). Here, a breeding simulator is regarded as a function whose input is a set of allocation parameters, and whose output is the final genetic gain. This figure is adapted from the Figure 1 in Hamazaki and Iwata (2024).

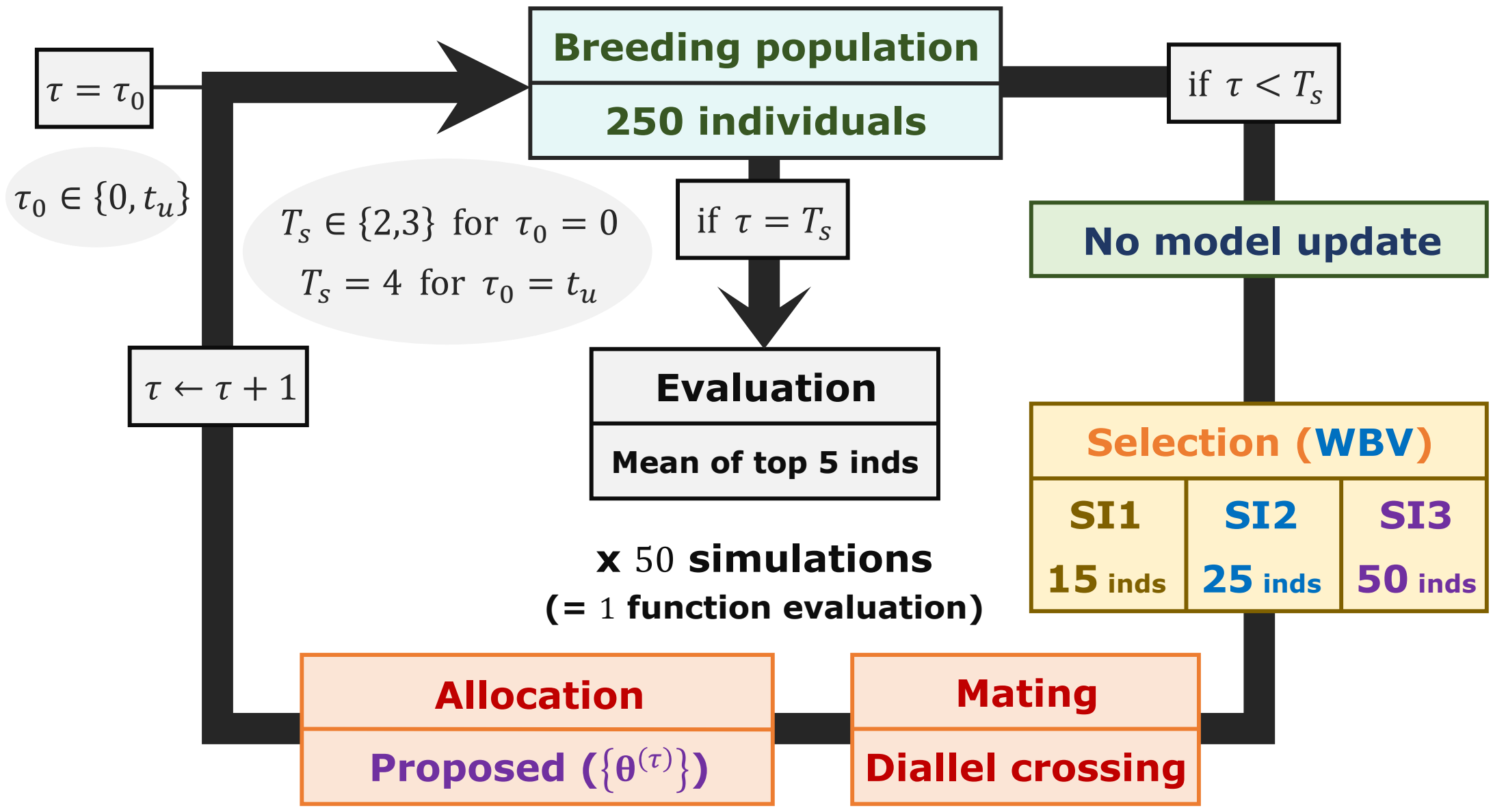

Supplemental Fig. 2. Images of breeding simulations conducted by the AI breeder used for the optimization of allocation strategies in ORA variants. Inside breeding simulations conducted by the AI breeder, there was no model update.

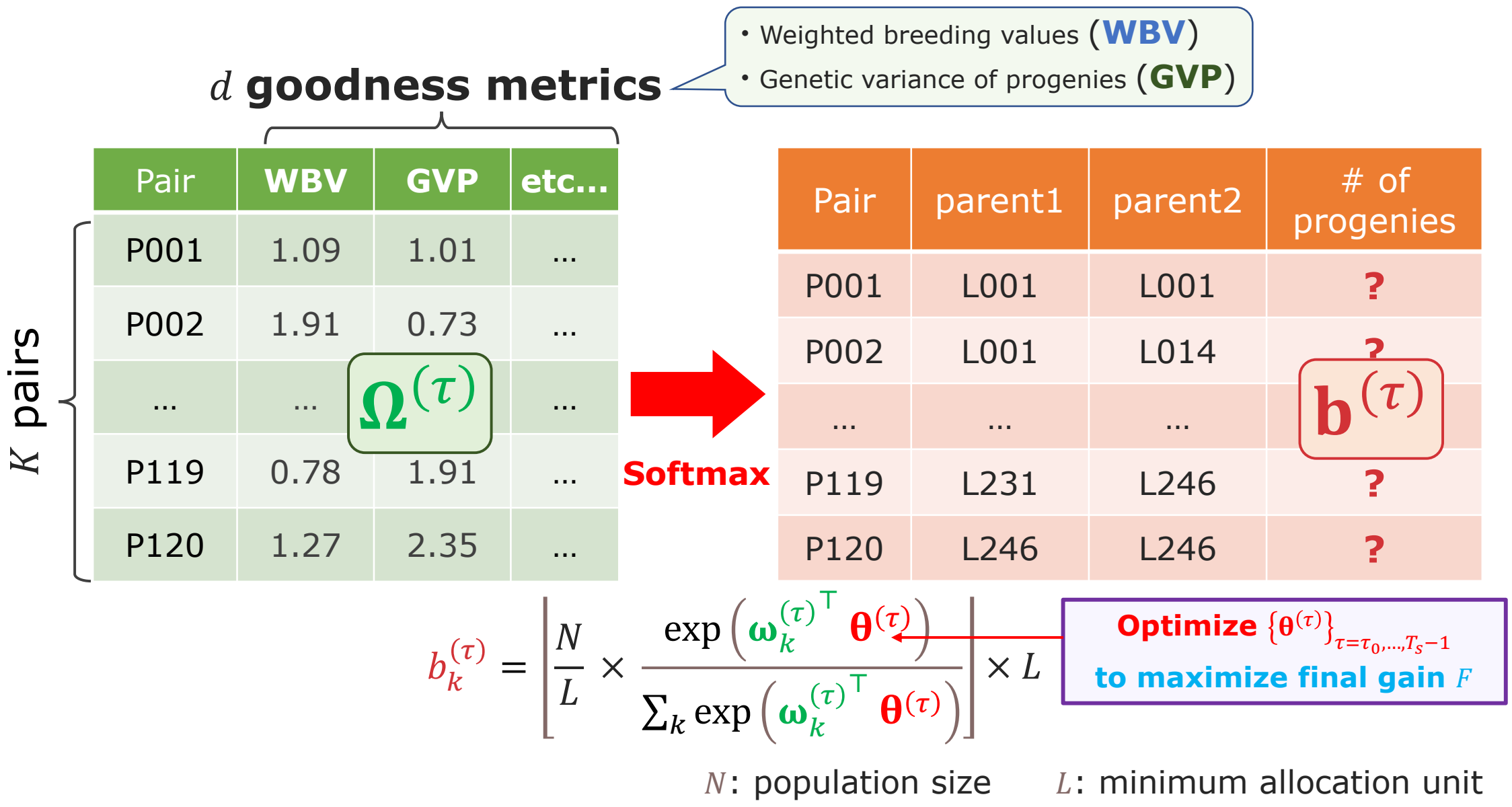

$$b_k^{(\tau)} = \left\lfloor \frac{N}{L} \times \frac{\exp\left({\boldsymbol{\omega}_k^{(\tau)}}^{\mathsf{T}} \boldsymbol{\theta}^{(\tau)}\right)}{\sum_k \exp\left({\boldsymbol{\omega}_k^{(\tau)}}^{\mathsf{T}} \boldsymbol{\theta}^{(\tau)}\right)} \right\rfloor \times L$$

Supplemental Fig. 3. The resource allocation strategy of progenies used in this study. After determining mating pairs by the diallel crossing between the parent candidates, given the parameter $\boldsymbol{\theta}^{(\tau)}$, the breeder automatically determines the number of progenies allocated to each pair $b_k^{(\tau)}$. This step is realized by utilizing the matrix $\boldsymbol{\Omega}^{(\tau)}$ consisting of features representing some goodness of mating pairs, i.e., WBV and GVP in this study. In other words, we assumed that $b_k^{(\tau)}$ is determined by applying the softmax function to the weighted sum of the multiple features. This figure is adapted from the Figure 2 in Hamazaki and Iwata (2024).

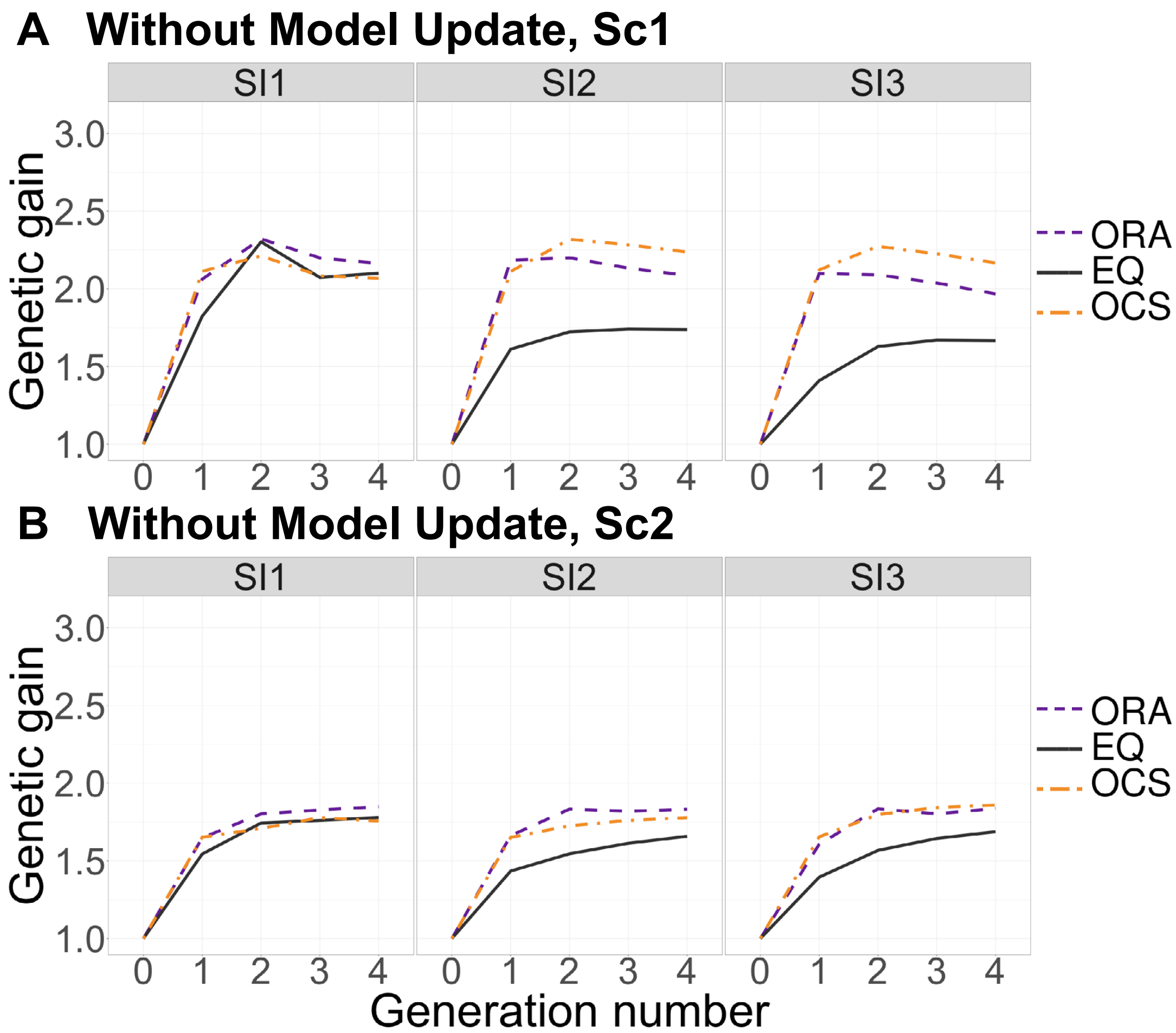

Supplemental Fig. 4. Change in the expected genetic gains over four generations under different selection intensities for the scheme without model updates. The horizontal and vertical axes represent the number of generations and the genetic gains, respectively. We compared the following three allocation strategies under the different selection intensities (SI1 – SI3): ORA: optimized resource allocation (purple dashed), EQ: equal allocation (black solid). OCS: optimal cross selection (orange dash-dotted line). Panel (A) corresponds to Scenario 1, while panel (B) corresponds to Scenario 2.

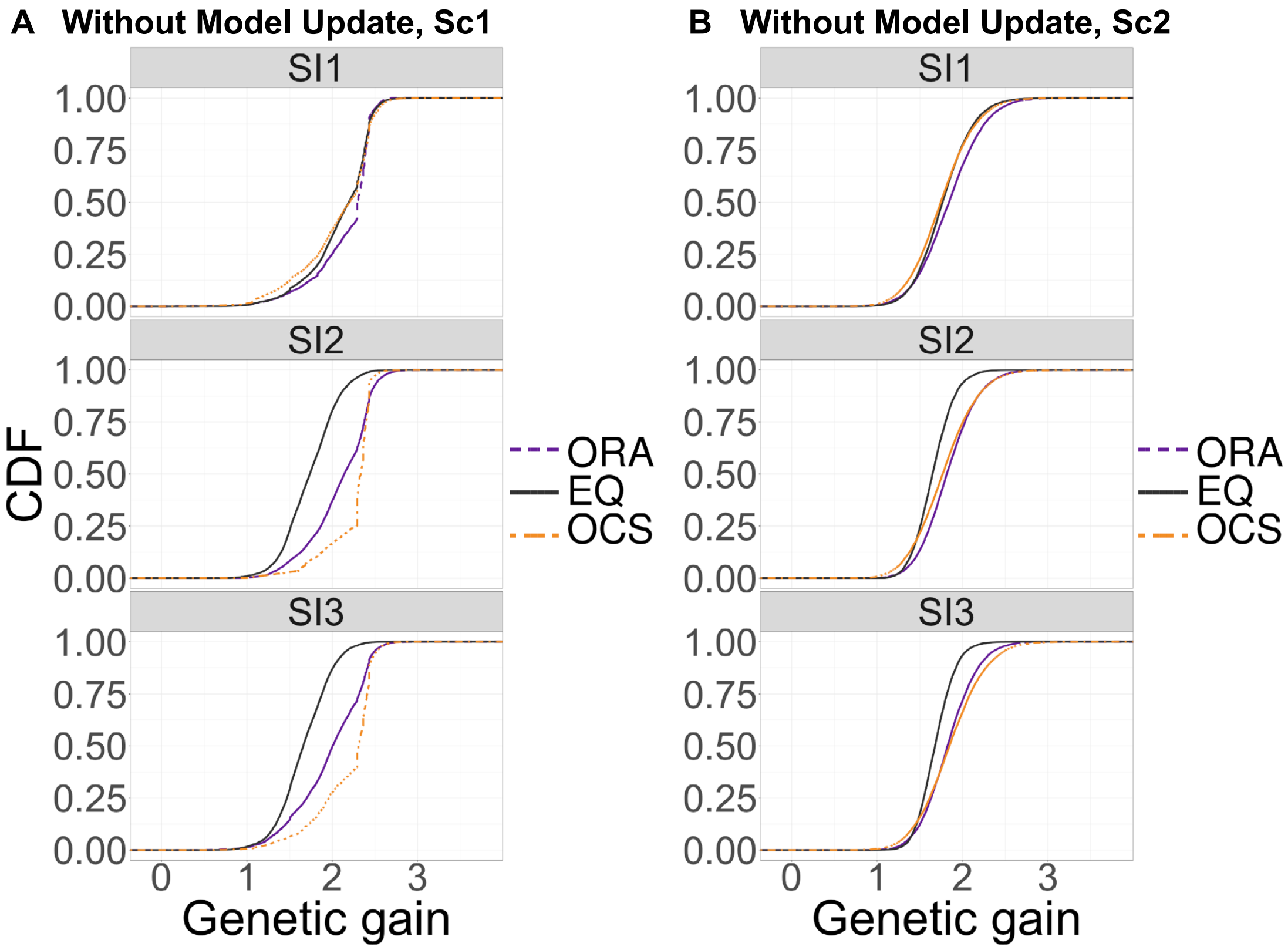

Supplemental Fig. 5. Genetic gains across different simulation repetitions for the scheme without model updates. The horizontal axis shows the final genetic gain of an individual, whereas the vertical axis represents the percentile of the simulation repetitions. Panel (A) corresponds to Scenario 1, while panel (B) corresponds to Scenario 2. The abbreviations of the allocation strategies are the same as those of Supplemental Fig. 4.

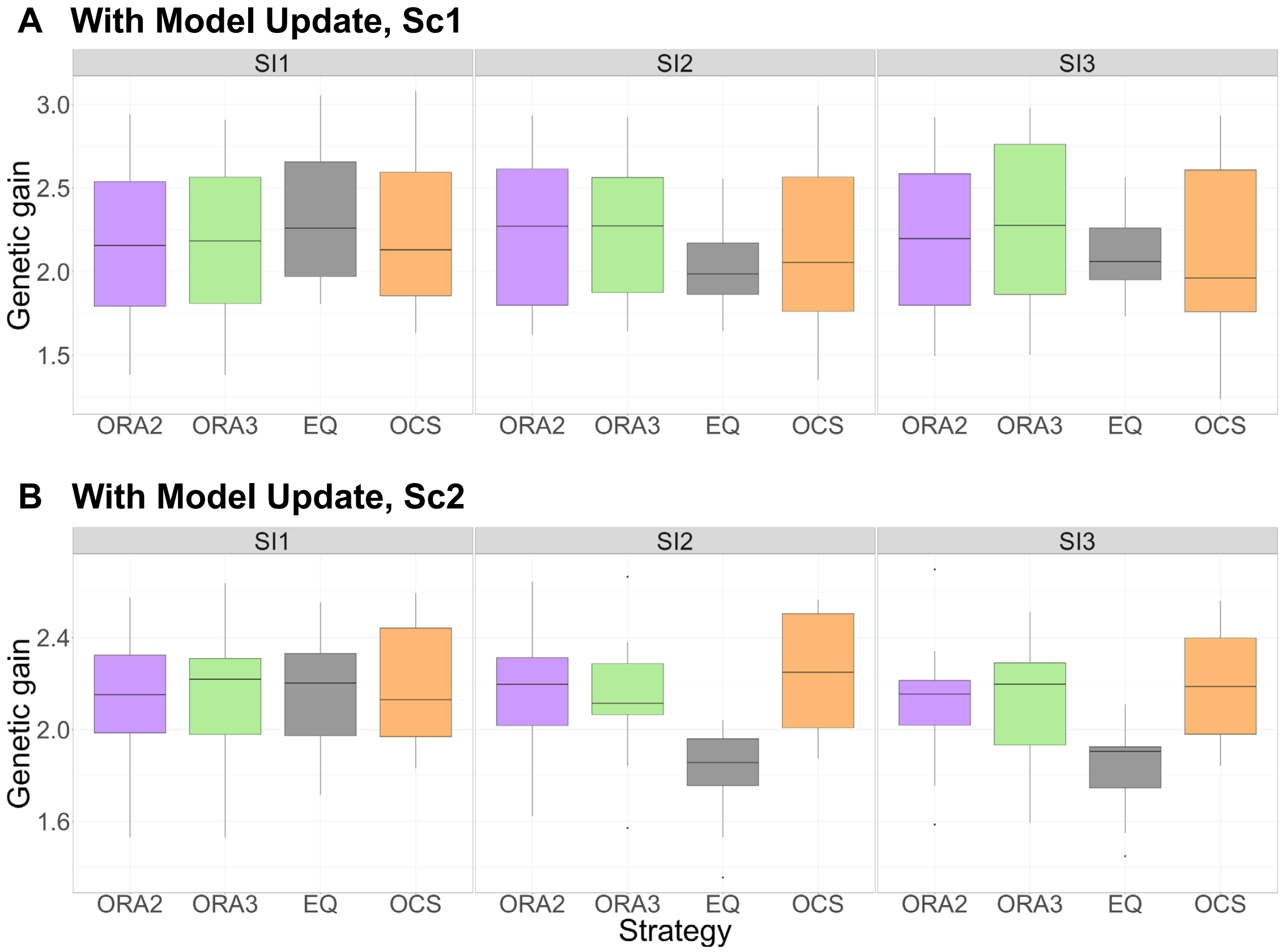

Supplemental Fig. 6. Genetic gains in the final generation using ten replications for the phenotype simulation under different selection intensities for the scheme with model updates. This figure includes boxplots of the genetic gains in the final generation using ten replications for the phenotype simulation. The horizontal and vertical axes represent the different allocation strategies and the final genetic gains, respectively. We compared the following allocation strategies under the different selection intensities (SI1 – SI3): ORA2 (purple), ORA3 (light green), EQ (grey), and OCS (orange), with details given in Fig. 3. Panel (A) corresponds to Scenario 1, while panel (B) corresponds to Scenario 2.

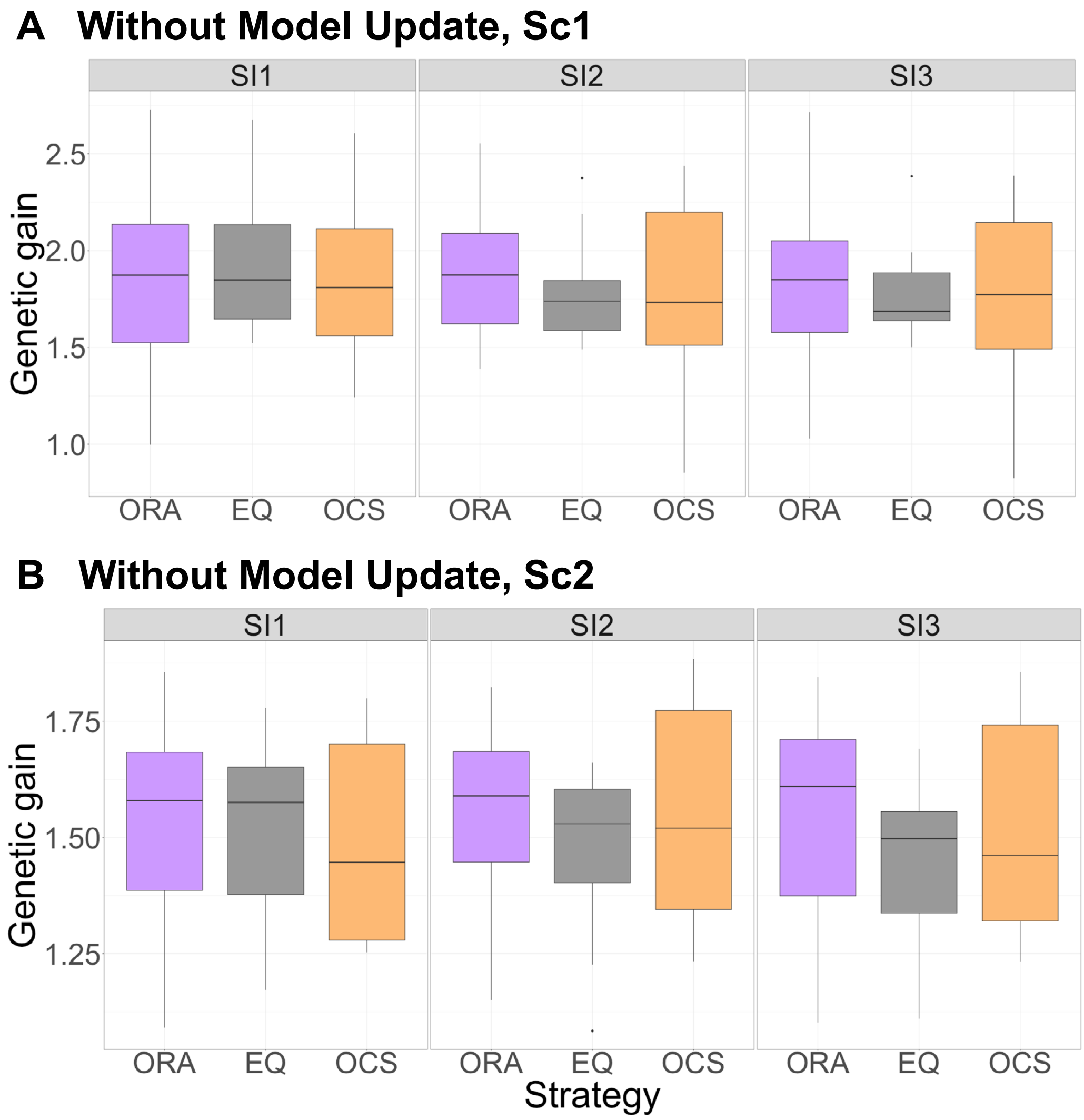

Supplemental Fig. 7. Genetic gains in the final generation using ten replications for the phenotype simulation under different selection intensities for the scheme without model updates. This figure includes boxplots of the genetic gains in the final generation using ten replications for the phenotype simulation. The horizontal and vertical axes represent the different allocation strategies and the final genetic gains, respectively. We compared the following allocation strategies under the different selection intensities (SI1 – SI3): ORA (purple), EQ (grey), and OCS (orange), with details given in Supplemental Fig. 4.

Panel (A) corresponds to Scenario 1, while panel (B) corresponds to Scenario 2.

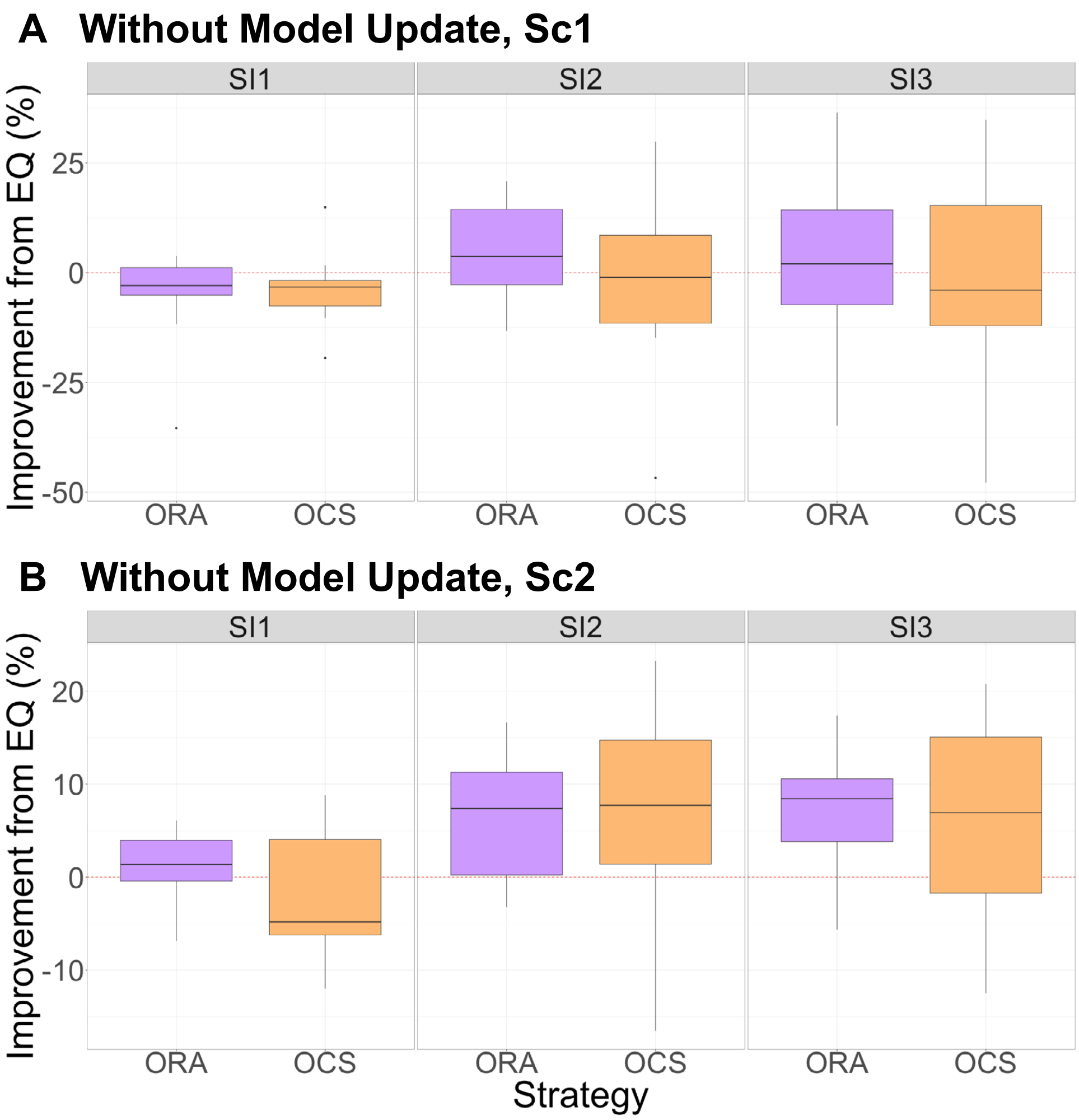

Supplemental Fig. 8. Improvement of the final genetic gains compared to the equal allocation strategy using ten replications for the phenotype simulation under different selection intensities for the scheme without model updates. This figure includes boxplots of the improvement of the final genetic gains compared to the equal allocation strategy using ten replications for the phenotype simulation. The horizontal and vertical axes represent the different allocation strategies and the improvement rate of the final genetic gains, respectively. We compared the following allocation strategies under the different selection intensities (SI1 – SI3): ORA (purple) and OCS (orange), with details given in

Supplemental Fig. 4. Panel (A) corresponds to Scenario 1, while panel (B) corresponds to Scenario 2.

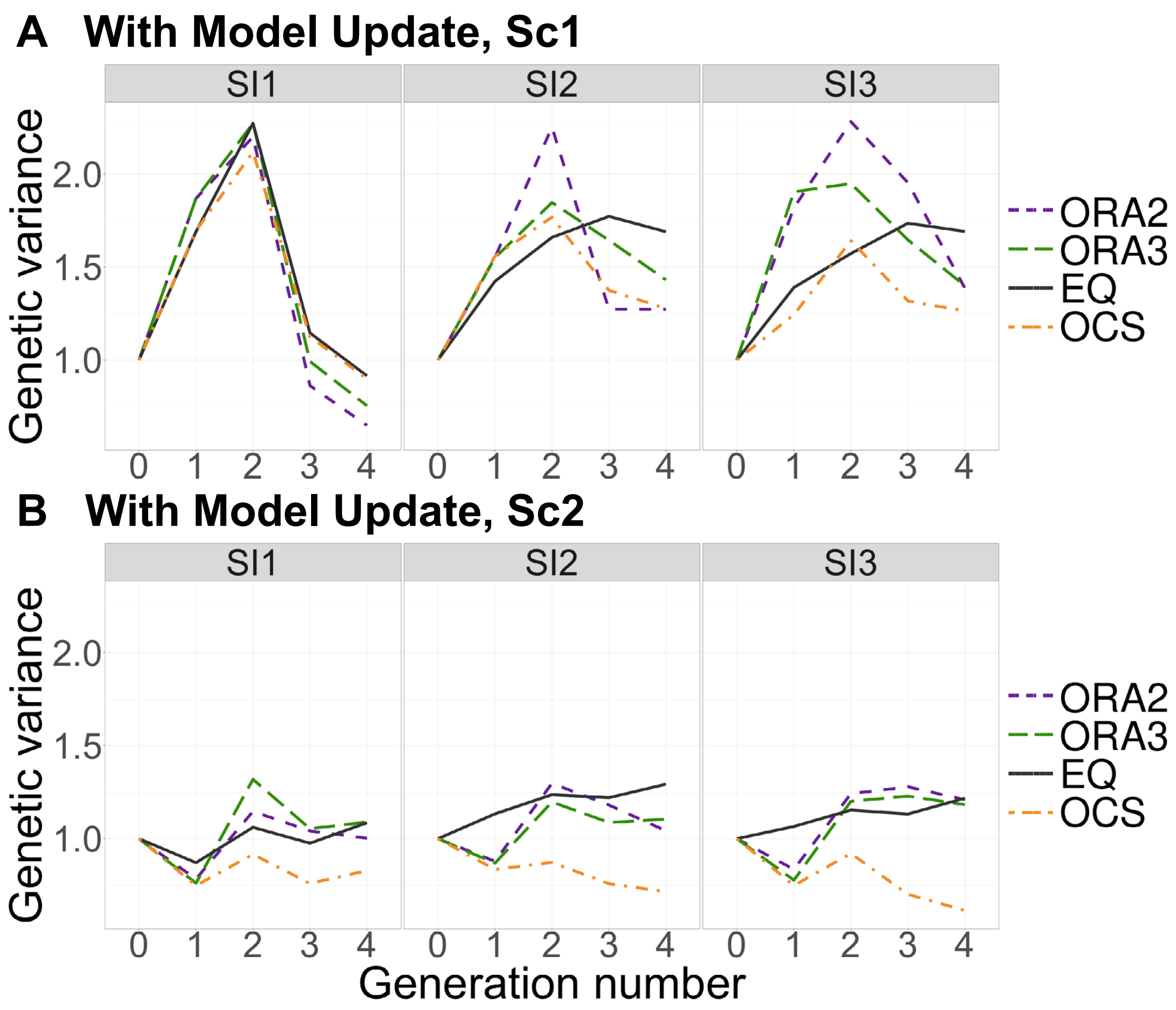

Supplemental Fig. 9. Change in the genetic variances over four generations under different selection intensities for the scheme with model updates. The horizontal and vertical axes represent the number of generations and the genetic variances in the breeding populations, respectively. Panel (A) corresponds to Scenario 1, while panel (B) corresponds to Scenario 2. The abbreviations of the allocation strategies are the same as those of Fig. 3.

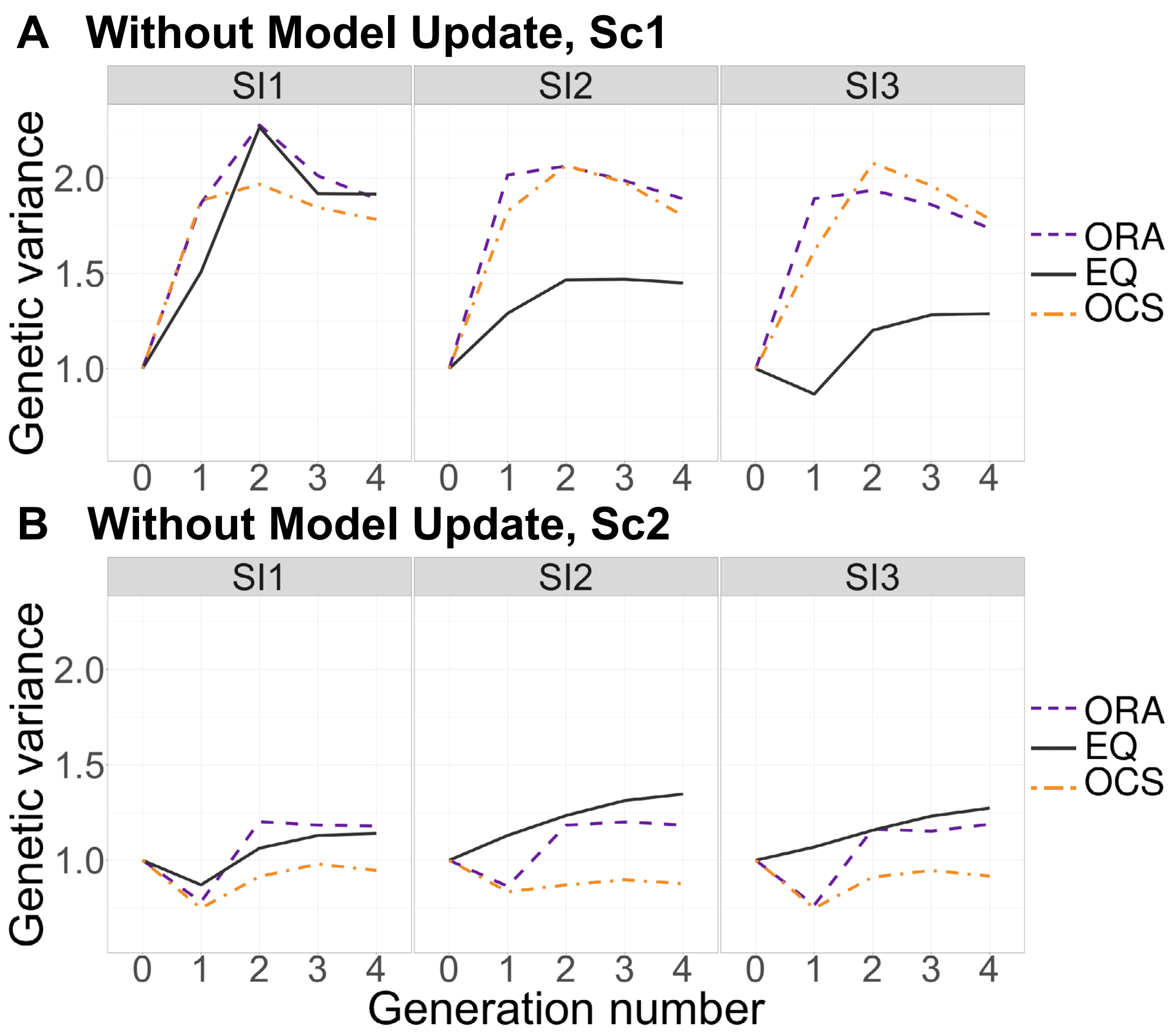

Supplemental Fig. 10. Change in the genetic variances over four generations under different selection intensities for the scheme without model updates. The horizontal and vertical axes represent the number of generations and the genetic variances in the breeding populations, respectively. Panel (A) corresponds to Scenario 1, while panel (B) corresponds to Scenario 2. The abbreviations of the allocation strategies are the same as those of Supplemental Fig. 4.

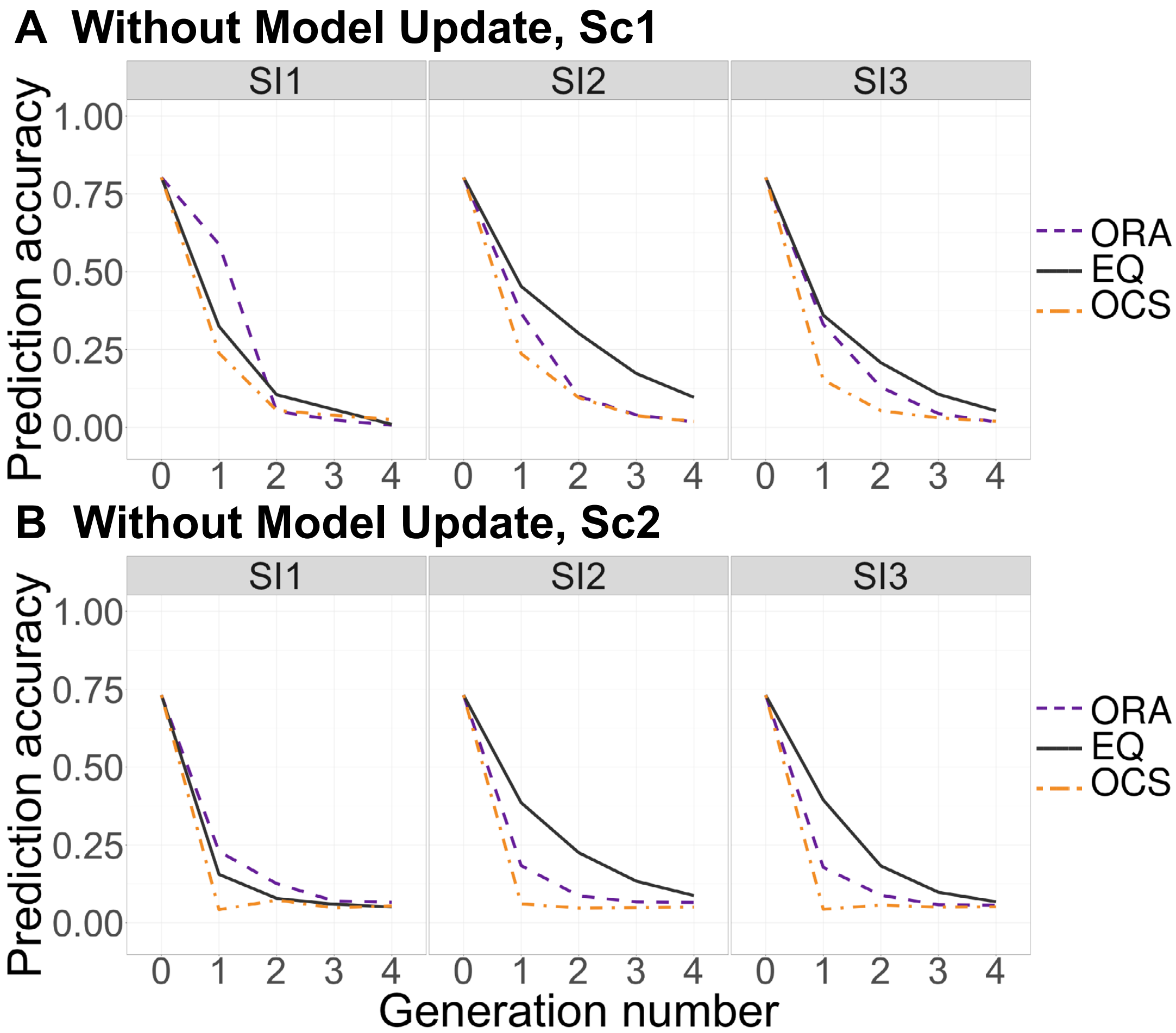

Supplemental Fig. 11. Change in the prediction accuracies over four generations under different selection intensities for the scheme without model updates. The horizontal and vertical axes represent the number of generations and the prediction accuracies for the breeding populations, respectively. Panel (A) corresponds to Scenario 1, while panel (B) corresponds to Scenario 2. The abbreviations of the allocation strategies are the same as those of Supplemental Fig. 4.
