# Supplementary material for: Optimizing progeny allocation strategies in breeding schemes while updating genomic prediction models
Source: Breed Sci. 2026 Jun 25;76(3):285–98. doi: 10.1270/jsbbs.25056 (PMC13709625; doi:10.1270/jsbbs.25056)
Supplement: Supplementary file 2 [file 76_285_s2.pdf]

doi: 10.1270/jsbbs.25056

**Supplemental Table 1.** Glossary table for the variables in the Methods & Materials section in the main manuscript

| Variable | Space | Explanation |
|---|---|---|
| $\boldsymbol{\tau}$ | $\{\tau_0, \dots, T_s - 1\}$ | current generation number in breeding simulations |
| $\boldsymbol{\tau_0}$ | $\mathbb{N}$ | initial generation number in breeding simulations |
| $\boldsymbol{T_s}$ | $\mathbb{N}$ | final generation number in breeding simulations |
| $\boldsymbol{t}$ | $\{0, \dots, T_r - 1\}$ | current generation number in actual breeding schemes |
| $\boldsymbol{T_r}$ | $\mathbb{N}$ | final generation number in actual breeding schemes |
| $\boldsymbol{t_u}$ | $0 < t_u < T_r$ | generation number where the GP model is updated |
| $\boldsymbol{\theta}^{(\tau)}$ | $\mathbb{R}^d$ | allocation-related parameter vector to be optimized in each generation $\tau$ |
| $\boldsymbol{\eta}^{(\tau)}$ | $\mathbb{R}^M$ | random factors related to recombination events when generating new individuals from mating pairs in each generation $\tau$ |
| $\boldsymbol{g}^{(T_s)}$ | $\mathbb{R}$ | the improvement in genotypic values of the top 5 % of individuals in the final population compared to the initial population |
| $\boldsymbol{N}$ | $\mathbb{N}$ | population size (constant across generations) |
| $\boldsymbol{q}$ | $\mathbb{N}$ | the number of QTLs per chromosome |
| $\boldsymbol{M}$ | $\mathbb{N}$ | the number of markers in total |
| $\mathbf{y}^{(t)}$ | $\mathbb{R}^N$ | vector of phenotypic values at generation $t$ |
| $\mathbf{1}$ | $\mathbb{R}^N$ | vector that vertically aligns 1 |
| $\boldsymbol{\mu}$ | $\mathbb{R}$ | intercept |
| $\mathbf{W}^{(t)}$ | $\{0,1,2\}^{N \times M}$ | matrix of marker genotype scores at generation $t$ |
| $\mathbf{w}_m^{(t)}$ | $\{0,1,2\}^N$ | vector of genotype scores at marker $m$ in generation $t$ |
| $\boldsymbol{\alpha}$ | $\mathbb{R}^M$ | vector of marker effects |
| $\hat{\boldsymbol{\alpha}}_m$ | $\mathbb{R}$ | marker effect at marker $m$ |
| $\mathbf{e}^{(t)}$ | $\mathbb{R}^N$ | error term for the GP model |
| $\mathbf{u}^{(\mathbf{WBV})(t)}$ | $\mathbb{R}^N$ | vector of of weighted breeding values at generation $t$ |
| $\boldsymbol{u}_{k_i}^{(\mathbf{WBV})(\tau)}$ | $\mathbb{R}$ | weighted breeding values of $k_i$-th individual at generation $\tau$ |
| $\mathbf{u}^{(t)}$ | $\mathbb{R}^N$ | vector of breeding values at generation $t$ |
| $\boldsymbol{p}_m^{(t)}$ | $\mathbb{R}$ | minor allele frequency at marker $m$ in generation $t$ |
| $\boldsymbol{n}$ | $\mathbb{N}$ | the number of selected genotypes as parent candidates |

| | | |
|---|---|---|
| $\boldsymbol{K}$ | $\mathbb{N}$ | the number of pair candidates |
| $\boldsymbol{L}$ | $\mathbb{N}$ | the minimum number of progenies that must be allocated to each pair |
| $\boldsymbol{b}_{\boldsymbol{k}}^{(t)}$ | $\mathbb{N}$ | the number of progenies allocated to each pair candidate $k$ at generation $t$ |
| $\boldsymbol{K}^{'}$ | $\mathbb{N}$ | the maximum number of pairs for the EQ strategy |
| $\boldsymbol{K}^{''}$ | $\mathbb{N}$ | the number of selected pairs for the OCS strategy |
| $\widetilde{\boldsymbol{u}}^{(\mathbf{top})(t)}$ | $\mathbb{R}$ | empirical mean of $\mathbf{u}^{(t)}$ for the top five genotypes |
| $\boldsymbol{u}^{(\mathbf{mean})(\mathbf{0})}$ | $\mathbb{R}$ | initial population's mean of breeding values |
| $\boldsymbol{\sigma}_{\boldsymbol{u}}^{(\mathbf{0})}$ | $\mathbb{R}$ | initial population's standard deviation of breeding values |
| $\boldsymbol{u}^{(\mathbf{top})(t)}$ | $\mathbb{R}$ | scaled empirical mean of $\mathbf{u}^{(t)}$ for the top five genotypes |
| $\boldsymbol{\omega}_{\boldsymbol{k}}^{(\tau)}$ | $\mathbb{R}^{d}$ | feature vector of goodness metrics |
| $\boldsymbol{k}_{\mathbf{MAT}}$, $\boldsymbol{k}_{\mathbf{PAT}}$ | $\mathbb{N}$ | indices of maternal and paternal parents |
| $\boldsymbol{\sigma}_{\boldsymbol{kmm}^{'}}^{(\tau)}$ | $\mathbb{R}$ | covariance between markers $m$ and $m^{'}$ resulting from progeny segregation derived from pair $k$ at generation $\tau$ |
