# Supplementary material for: Optimizing progeny allocation strategies in breeding schemes while updating genomic prediction models
Source: Breed Sci. 2026 Jun 25;76(3):285–98. doi: 10.1270/jsbbs.25056 (PMC13709625; doi:10.1270/jsbbs.25056)
Supplement: Supplementary file 3 [file 76_285_s3.pdf]

Supplement Text 1

Details of Optimal Cross Selection

OCS selects superior crossing pairs from all the possible combinations in the current population, considering both the GEBVs and genetic diversity of the selected individuals (Gorjanc et al. 2018, Allier et al. 2019). In this study, we implemented OCS following the method in Sakurai et al. (2024). This method solves the following optimization problem to determine the selected pairs.

$$\mathrm{argmax}_{a_k} \sum_{k=1}^{K} a_k \bar{u}_k^{(\mathrm{BV})(t)} \ , \qquad \text{(S1)}$$

$$\mathrm{s.t.}\ \gamma^{(t)} > h^{(t)}, \qquad \text{(S2)}$$

where $K = \frac{n(n+1)}{2}$ is the number of total mating pairs, $a_k \in \{0,1\}$ is a dummy variable that indicates whether pair k is selected ($a_k = 1$) of not ($a_k = 0$), and $\bar{u}_k^{(\mathrm{BV})(t)} \in \mathbb{R}$ is the mean of the two parents' GEBVs ($\bar{u}_k^{(\mathrm{BV})(t)} = \frac{1}{2}\sum_{i\in\{\mathrm{MAT,PAT}\}} \mathbf{w}_{k_i}^{(t)\top} \hat{\boldsymbol{\alpha}}$, where $\mathbf{w}_{k_i}^{(t)} \in \{0,1,2\}^M$ is a vector of genotype scores of individual k_i in generation t and $\hat{\boldsymbol{\alpha}} \in \mathbb{R}^M$ is a vector of estimated marker effects). $\gamma^{(t)} \in \mathbb{R}$ represents the genetic diversity of all selected mating pairs, while $h^{(t)} \in \mathbb{R}$ is the genetic diversity constraint at generation t. First, $\gamma^{(t)}$ is defined as follows in Equation S3.

$$\gamma^{(t)} = 1 - \mathbf{c}^\top \mathbf{K}^{(t)} \mathbf{c}. \qquad \text{(S3)}$$

Here, $\mathbf{c} \in \{0,1\}^n$ is a contribution vetor to each individual, computed as $\mathbf{c} = \frac{1}{2K''}\mathbf{Za}$, where $\mathbf{Z} \in \{0,1\}^{n \times K}$ is a design matrix indicating the correspondence between parent candidates and pairs, $\mathbf{a} = [a_1, \dots, a_K]^\top$, and $K'' = 25$ is the number of pairs selected by OCS. $\mathbf{K}^{(t)} \in \mathbb{R}^{n \times n}$ is an identical-by-state matrix, which can be computed as shown in Equation S4.

$$\mathbf{K}^{(t)} = \frac{1}{2}\left(\frac{1}{M}\left(\mathbf{W}^{(t)} - 1\right)\left(\mathbf{W}^{(t)} - 1\right)^\top + 1\right), \tag{S4}$$

where $\mathbf{W}^{(t)} \in \{0,1,2\}^{N \times M}$ is a matrix of marker genotype scores at generation t as in the main manuscript.

Next, $h^{(t)}$ is defined as shown in Equation S5, which was originally proposed by Allier et al. (2019).

$$h^{(t)} = \begin{cases} h^{(0)} + \left(\frac{t}{t^\star}\right)^s \left(h^\star - h^{(0)}\right) \text{ for } t \le t^\star \\ h^\star \text{ for } t > t^\star \end{cases}, \tag{S5}$$

Here, $h^{(0)}$ is the mean heterozygosity rate of the initial population, and $t^\star \in \mathbb{N}$, $h^\star \in \mathbb{R}$, and $s \in \mathbb{R}$ are hyperparameters that define the OCS. $h^{(0)}$ is computed as shown in the following Equation S6.

$$h^{(0)} = \frac{1}{M}\sum_{m=1}^{M} 2p_m^{(0)}\left(1 - p_m^{(0)}\right), \tag{S6}$$

where $p_m^{(0)} \in \mathbb{R}$ is the minor allele frequency at marker m in generation 0, as defined in Equation 4 in the main manuscript. As for the hyperparameters in OCS, $t^\star$ represents

the target generation, $h^\star$ indicates the genetic diversity to be maintained in the target generation, and s is the shape parameter that defines the trajectory of $h^{(t)}$. In this study, we set $t^\star = T_r = 4$, $h^\star = 0.01h^{(0)}$, and $s = 1$.
